# Bacterial SBP56 identified as a Cu-dependent methanethiol oxidase widely distributed in the biosphere

**DOI:** 10.1038/ismej.2017.148

**Published:** 2017-10-24

**Authors:** Özge Eyice, Nataliia Myronova, Arjan Pol, Ornella Carrión, Jonathan D Todd, Tom J Smith, Stephen J Gurman, Adam Cuthbertson, Sophie Mazard, Monique ASH Mennink-Kersten, Timothy DH Bugg, Karl Kristoffer Andersson, Andrew WB Johnston, Huub JM Op den Camp, Hendrik Schäfer

**Affiliations:** 1 School of Life Sciences, University of Warwick, Coventry, UK; 2 School of Biological and Chemical Sciences, Queen Mary University of London, London, UK; 3 Department of Microbiology, Institute for Water and Wetland Research, Faculty of Science, Radboud University, Nijmegen, The Netherlands; 4 School of Biological Sciences, University of East Anglia, Norwich, UK; 5 Department of Biosciences and Chemistry, Sheffield Hallam University, Sheffield, UK; 6 Department of Physics and Astronomy, University of Leicester, Leicester, UK; 7 Department of Chemistry, University of Warwick, Coventry, UK; 8 Department of Bioscience, University of Oslo, Oslo, Norway

## Abstract

Oxidation of methanethiol (MT) is a significant step in the sulfur cycle. MT is an intermediate of metabolism of globally significant organosulfur compounds including dimethylsulfoniopropionate (DMSP) and dimethylsulfide (DMS), which have key roles in marine carbon and sulfur cycling. In aerobic bacteria, MT is degraded by a MT oxidase (MTO). The enzymatic and genetic basis of MT oxidation have remained poorly characterized. Here, we identify for the first time the MTO enzyme and its encoding gene (*mtoX*) in the DMS-degrading bacterium *Hyphomicrobium* sp. VS. We show that MTO is a homotetrameric metalloenzyme that requires Cu for enzyme activity. MTO is predicted to be a soluble periplasmic enzyme and a member of a distinct clade of the Selenium-binding protein (SBP56) family for which no function has been reported. Genes orthologous to *mtoX* exist in many bacteria able to degrade DMS, other one-carbon compounds or DMSP, notably in the marine model organism *Ruegeria pomeroyi* DSS-3, a member of the Rhodobacteraceae family that is abundant in marine environments. Marker exchange mutagenesis of *mtoX* disrupted the ability of *R. pomeroyi* to metabolize MT confirming its function in this DMSP-degrading bacterium. In *R. pomeroyi*, transcription of *mtoX* was enhanced by DMSP, methylmercaptopropionate and MT. Rates of MT degradation increased after pre-incubation of the wild-type strain with MT. The detection of *mtoX* orthologs in diverse bacteria, environmental samples and its abundance in a range of metagenomic data sets point to this enzyme being widely distributed in the environment and having a key role in global sulfur cycling.

## Introduction

**Figure 1:**
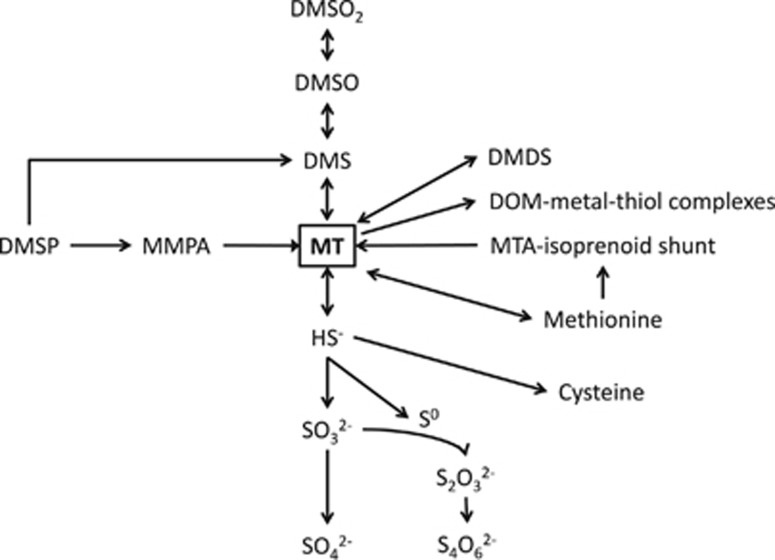
Simplified schematic showing the role of MT as an intermediate in the metabolism of sulfur compounds. A single arrow does not imply a single biotransformation step. DMDS, dimethyldisulfide; DMSO, dimethylsulfoxide; DMSO_2_, dimethylsulfone; DOM, dissolved organic matter; HS^−^, sulfide ion; MTA, 5′-methylthioadenosine; SO_3_^2−^, sulfite ion; S^0^, elemental sulfur; S_2_O_3_^2−^, thiosulfate; S_4_O_6_^2−^, tetrathionate; SO_4_^2−^, sulfate.

Methanethiol (CH_3_SH; methylmercaptan, MT) is a foul-smelling gas with a low odor threshold. As a malodorous compound that can be detected by the human nose at very low concentration (odor threshold 1–2 p.p.b., (Devos *et al.*, 1990)), it has a significant role in causing off-flavors in foods and beverages and it is one of the main volatile sulfur compounds causing halitosis in humans (Awano *et al.*, 2004; Tangerman and Winkel, 2007). The production and degradation of MT are major steps in the biogeochemical cycle of sulfur (Figure 1). Sources of MT include the methylation of sulfide in anoxic habitats, demethiolation of sulfhydryl groups and degradation of sulfur-containing amino acids (Lomans *et al.*, 2001, 2002; Bentley and Chasteen, 2004). MT is produced in the marine environment as an intermediate of dimethylsulfoniopropionate (DMSP) degradation by the demethylation pathway. In this pathway, initial demethylation of DMSP to methylmercaptopropionic acid (MMPA) is carried out by the DMSP-dependent demethylase (DmdA) (Howard *et al.*, 2006). Subsequent degradation of MMPA occurs via MMPA-CoA to methylthioacryloyl-CoA and then to acetaldehyde and MT by the enzymes DmdB, DmdC and DmdD, respectively (Reisch *et al.*, 2011b). MT is also produced as an intermediate of dimethylsulfide (DMS) degradation (Lomans *et al.*, 1999a, 2002; Bentley and Chasteen, 2004; Schäfer *et al.*, 2010).

Only few measurements of MT in the environment have been reported. Analysis of volatile sulfur compounds in freshwater ditches demonstrated that MT was the dominant volatile organic sulfur compound reaching concentrations of 3–76 nm in sediments and 1–8 nm in surface freshwater (Lomans *et al.*, 1997). Measurements of MT concentrations in the surface ocean water are scarce. Studies reporting MT measurements in seawater suggest a typical range of ~0.02–2 nm (Ulshöfer *et al.*, 1996; Kettle *et al.*, 2001; Xu *et al.*, 2001).

Microbial uptake and degradation of MT are important sinks for MT. Despite low MT concentrations in seawater, radiotracer experiments showed that trace levels of MT (0.5 nm) were rapidly taken up and incorporated into biomass by marine bacterioplankton (Kiene *et al.*, 1999). Besides this assimilation, MT degradation through its utilization as a carbon and energy source in methanogenic archaea, sulfate-reducing bacteria, and aerobic bacteria (Lomans *et al.*, 1999b, 2001, 2002; Schäfer *et al.*, 2010) and its methylation to DMS by the recently described methyltransferase MddA (MddA: methanethiol-dependent DMS) (Carrión *et al.*, 2015) contribute to biological MT removal.

The molecular basis of MT degradation remains poorly understood. In aerobic sulfur-oxidizing and methylotrophic bacteria including strains of *Thiobacillus* (Gould and Kanagawa, 1992; Lee *et al.*, 2002), *Rhodococcus* (Kim *et al.*, 2000) and *Hyphomicrobium* (Suylen *et al.*, 1987), MT is degraded by a MT oxidase (MTO) to formaldehyde, hydrogen sulfide and hydrogen peroxide; however, inconsistent data have emerged from these studies. Estimated molecular weights of MTOs characterized previously have ranged from ~29–61 kDa. The MTO from *Hyphomicrobium* sp. EG was reported to be a monomer of 40–50 kDa that was insensitive to metal-chelating agents (Suylen *et al.*, 1987). In *Thiobacillus thioparus* (Gould and Kanagawa, 1992), MTO also appeared to be a monomer with a molecular weight of ~40 kDa; however, a later study of MTO in *T. thioparus* reported a different molecular weight for MTO of 61 kDa (Lee *et al.*, 2002). MTO from *Rhodococcus rhodochrous* was reported to have a molecular weight of 64.5 kDa (Kim *et al.*, 2000). The genetic basis of MT degradation has not been identified, constituting a gap in fundamental knowledge of a key step in the global sulfur cycle.

Here, we report new insights into the biochemistry, genetics and environmental distribution of methanethiol oxidases in bacteria. We purified and characterized MTO from *Hyphomicrobium* sp. VS a DMS-degrading methylotrophic bacterium that was isolated from activated sewage sludge and which has MTO activity during growth on DMS as a sole carbon and energy source (Pol *et al.*, 1994). We identified the gene encoding MTO, *mtoX*, in *Hyphomicrobium* sp. VS and detected orthologous *mtoX* genes in a wide range of bacteria including methylotrophic, sulfur-oxidizing and DMSP-degrading bacteria. We then genetically analyzed its function and transcriptional regulation in a model isolate of the Rhodobacteraceae family, *Ruegeria pomeroyi* DSS-3, which produces MT during degradation of DMSP by the demethylation pathway (Reisch *et al.*, 2011a). The development of *mtoX*-specific PCR primers allowed testing environmental samples for the presence of *mtoX*-containing populations. This analysis suggested that the genetic potential of MT degradation is present in a wider spectrum of phylogenetic lineages than previously realized based on bacterial cultures. This was also reflected by the presence of *mtoX* genes from uncultivated organisms in diverse habitats based on screening of metagenomic data sets, which suggests that MTO is widely distributed in the biosphere.

## Materials and methods

### Growth of *Hyphomicrobium* sp. VS

*Hyphomicrobium* sp. VS was grown in continuous culture in a Fermac 300 series fermenter (Electrolabs, Tewkesbury, UK) as described previously (Boden *et al.*, 2011) using PV mineral medium using either DMS (12 mm) as sole substrate or in combination with methanol (both substrates 12 mm). The culture was held at 30 °C, aerated with sterile air at 1.5 l/min, and stirred at 200 r.p.m. pH was adjusted to 7.4±0.1 by automatic titration with 1 M NaOH. *Hyphomicrobium* sp. VS was initially grown for 24 h in a 1 liter volume in sterilized medium supplemented to 25 mm with methanol before beginning the addition of medium containing DMS. Overflow was collected in a vessel held on ice. Cells were collected daily, washed with 25 mm 1,4-piperazinediethanesulfonic acid (PIPES, pH 7.2) and resuspended in the same buffer. Concentrated cells were frozen in liquid nitrogen and stored at −80 °C.

### Protein purification and characterization

Thawed cells (~1.5 g dry weight) were washed with 25 mm PIPES (pH 7.2), centrifuged at 12 000 × *g* for 20 min at 4 °C and resuspended in 50 mm *N*-[Tris(hydroxymethyl)methyl]glycine (TRICINE, pH 8.2) supplemented with DNAse I (1 μg ml^−1^) and 1 mm benzamidine. A crude cell extract (~600 mg protein) was prepared by breaking the suspended cells using a Constant Cell Disrupter (Constant Systems, Daventry, UK) three times at 25 MPa and 4 **°**C. Unbroken cells and debris were removed by centrifugation in a Beckmann JA20 at 12 000 × *g* for 25 min at 4 **°**C followed by removal of membrane fractions by centrifugation of the supernatant at 144 000 × *g* for 90 min (BECKMAN rotor SW28, Beckman, Indianapolis, IN, USA). The final supernatant (~300 mg protein) was concentrated using an Amicon stirred cell with PM10 ultrafiltration membrane (Millipore, Watford, UK). Aliquots of concentrated supernatant (~10 mg ml^−1^; 0.5 ml) were applied to an anion-exchange MonoQ 10 column (GE Lifesciences, Little Chalfont, UK) equilibrated with precooled (4 °C) 10 mm TRICINE (pH 8.2) supplied with 1 mm benzamidine. An increasing (0–1 M) NaCl gradient was used to elute fractions, which were assayed for MTO activity (see below). Fractions with MTO activity were concentrated using an Amicon stirred cell with a PM10 ultrafiltration membrane. Concentrated MonoQ 10 fractions containing mainly MTO were subjected to gel filtration using a Superdex 75 column (GE Lifesciences) equilibrated in precooled (4 °C) 10 mm TRICINE (pH 8.2) supplied with 1 mm benzamidine. Fractions containing active MTO showed a single dominant polypeptide on SDS-PAGE and were collected and concentrated as described above before storage at −80 °C. Further detail about protein purification is given in Supplementary Tables S1 and S2. Analytical gel filtration was carried out using a Superdex 75 column equilibrated with 10 mm TRICINE, pH 8.2, 1 mm benzamidine, 0.15 M NaCl, at a flow rate of 1 ml min^−1^.

### MTO activity assays

Routine analysis of enzyme activity was carried out by measuring MT degradation using gas chromatography (GC) for which MT was analyzed in headspace samples (100 μl) using an Agilent gas chromatograph (Agilent Technologies, Cheshire, UK) fitted with a 30 m × 0.32 mm column (DB-1). Helium was used as the carrier gas at a temperature of 200 °C. The gas chromatograph had a flame ionization detector. Alternatively, MT was measured in headspace samples using a GC-2010plus (Shimadzu, Milton Keynes, UK) equipped with a Shim-1 column (30 m, 0.5 mm i.d.), at a temperature of 180 °C, with helium as carrier gas and a flame photometric detector. MTO activity was assayed in 10 mm TRICINE, pH 8.2 at 30 °C, typically using 0.1–0.5 mg of protein per assay. Alternatively, MTO activity was measured as substrate-induced O_2_ consumption in a Clark type oxygen electrode with and without addition of catalase (0.1 mg) and in the presence and absence of ZnSO_4_ (1 mm). The formation of formaldehyde by MTO was quantified using the Purpald reagent (Sigma-Aldrich, Gillingham, UK) as described previously (Boden *et al.*, 2011). Standard formaldehyde solutions were prepared from methanol-free formaldehyde in the range of 0–1 mm.

### Protein electrophoresis

SDS-PAGE electrophoresis was carried out using standard protocols using precast gels supplied by Bio-Rad (Hemel Hempstead, UK) run in 1 × Tris(hydroxymethyl)aminomethane (Tris) glycine buffer.

### Metal analysis

Quantification of various elements contained in purified MTO was performed using inductively coupled plasma (ICP) mass spectrometry at the ICI Measurement Science Group, Wilton, Middlesbrough, UK.

### Electron paramagnetic resonance spectroscopy

The electron paramagnetic resonance (EPR) spectral properties of MTO were examined under various reducing (5 mm ascorbate, 1 mm dithionite) and oxidizing conditions (1.8 mm sodium hexachloroiridate (V)), and under enzyme assay condition in the presence of substrate (all at 25 °C). All analyses were carried out with a preparation of MTO of 9.2 mg ml^−1^ in 10 mm Tricine, pH 8.2 (with 1 mm benzamidine) on a Bruker EleXsyS 560 SuperX spectrometer fitted with a Bruker ER41116DM dual mode cavity (Bruker Biospin, Rheinstetten, Germany) and an Oxford ESR 900 Helium Flow Cryostat (Oxford Instruments plc, Abingdon, UK). EPR spectra of oxidized MTO were recorded at temperatures of 7 and 13 K after addition of sodium hexachloroiridate (V) (1.8 mm final concentration) using a microwave frequency of 9.66 GHz, microwave power of 0.63 mW, a modulation amplitude of 7 Gauss and a time constant of 81 ms. Further EPR spectra (four scans) were also recorded in the presence of enzyme substrates ethanethiol (1 mm) and oxygen (0.2 mm similar to assay conditions) using instrument settings as detailed above, except for a microwave power of 0.2 mW, a modulation amplitude of 7.6 Gauss at a temperature of 15 K.

### X-ray spectroscopy analysis of methanethiol oxidase

X-ray absorption spectra were obtained in fluorescent mode on station B18 of the diamond light source (Harwell Science and Innovation Campus, Didcot, UK). This uses the technique of quick extended X-ray absorption fine structure (EXAFS), where the monochromator rotates at a constant rate during data acquisition. The fluorescence was detected using a nine-element germanium solid state detector. Data were obtained at the Cu K edge for a variety of samples and standards. All data were obtained with the samples at 77 K in a cryostat. To minimize radiation damage, the beam was rastered across the sample, which was moved between each scan. Each scan took about 20 min to acquire. Copper metal (foil), CuO and CuS were used as reference samples. The copper-containing enzyme tyrosinase (Sigma-Aldrich) was used as additional reference. Five samples of purified MTO were analyzed: as-isolated enzyme; enzyme treated with the oxidizing agent sodium hexachloroiridate (2 mm); enzyme treated with the substrate methanethiol; enzyme treated with the reducing agent sodium dithionite (1 mm). Detailed information about processing of data is provided in the Supplementary Information.

### Identification of the gene encoding MTO in *Hyphomicrobium* sp. VS

N-terminal sequence data for MTO were obtained from gel slices of Coomassie-stained SDS-PAGE gels by ALTA Bioscience, University of Birmingham, UK. Internal peptide sequences were determined by the biological mass spectrometry facility in the School of Life Sciences, University of Warwick, as described previously (Schäfer *et al.*, 2005). We sequenced genomic DNA of *Hyphomicrobium* sp. VS using Illumina technology. After quality trimming, 26 777 191 reads with an average length of 60 bp were obtained. Reads were assembled using a combination of the CLCBio (Aarhus, Denmark) and Edena assemblers (Hernandez *et al.*, 2014). No gap-closing was performed. This resulted in a draft genome consisting of 347 contigs (average length 9125 bp) with a total size of 3 722 323 bases (See Supplementary Table S3). Peptide sequences were matched against proteins predicted by the annotation pipeline. The draft genome assembly for *Hyphomicrobium* sp. VS is available on the MaGe Microscope platform at http://www.genoscope.cns.fr/agc/microscope/mage/index.php (Vallenet *et al.*, 2013). The sequence of the contig containing *mtoX*, *SCO1/senC* and *mauG* has been deposited with the National Center for Biotechnology Information under accession number KY242492.

### Phylogenetic analysis

Nucleic acid sequences were imported into Arb (Ludwig *et al.*, 2004) and translated before aligning using clustalx as implemented in Arb. A phylogenetic tree was derived using amino acid sequence data based on the Arb neighbor joining method, using alignment columns corresponding to positions 85–300 of the MtoX polypeptide of *Hyphomicrobium* sp. VS and the PAM (point accepted mutation) distance correction as implemented in Arb. Bootstrapping (100 iterations) was carried out in MEGA 5 (Tamura *et al.*, 2011).

### Genetic analysis of *mtoX* in *Ruegeria pomeroyi* DSS-3

Locus SPOA0269 was identified by blast search as a homolog of *mtoX* in *R. pomeroyi*. Two PCR primer pairs were designed to amplify the flanking regions of SPOA0269 (5′-GCGAATTCTCGAAGCCATCGCTGG-3′ with 5′-CGGGATCCCATCGCCAGGGCACCGG-3′ and 5′-CGGGATCCTGGGCCTGGGCCGCGCGC-3′ with 5′-CCCAAGCTTCGGGGTCCGCCGGGTCAGG-3′). The resulting PCR products were digested with *BamH*I ligated together to form a clone with a truncated version of SPOA0269 (2/3 deletion in frame of the gene). The resulting fragment was digested with *Eco*RI and *Hind*III and then cloned into pK18sac. Then, a spectinomycin resistance (Spec^R^) cassette was cloned into a unique *BamH*I site within the truncated version of the gene. This construct was transferred by tri-parental conjugational mating with *Escherichia coli* containing the mobilizing plasmid pRK2013 as the helper strain (Figurski and Helinski, 1979) into rifampicin resistant *R. pomeroyi* J470 (Todd *et al.*, 2011) (20 μg ml^−1^). Colonies were selected based on resistance to spectinomycin (200 μg ml^−1^) and sucrose (5%), but sensitivity to kanamycin (20 μg ml^−1^). Such colonies were checked by PCR and by southern blotting to show that they were mutated in SPOA0269.

### Enzymatic assays of MTO activity in *R. pomeroyi* DSS-3

For the measurement of MT consumption by *R. pomeroyi* whole cells, *R. pomeroyi* DSS-3 wild-type and *mtoX*^−^ strains were grown overnight at 28 °C in marine basal medium (MBM) (Baumann and Baumann, 1981) or MBM supplemented with 200 μg ml^−1^ spectinomycin, respectively, using succinate (10 mm) as a carbon source and NH_4_Cl (10 mm). Cultures were spun down and pellets were washed three times with fresh MBM. After that, cell suspensions were adjusted to an OD_600_=1.4 and inoculated (1/10 dilution) into 120 ml serum vials containing 20 ml MBM plus 0.5 mm MT. Vials were incubated at 28 °C and MT concentration in the headspace was measured at time 0 and after 6 h by GC as described in (Carrión *et al.* (2015)). Chemical degradation of MT in the medium control was subtracted from the MT removed in *R. pomeroyi* cultures to calculate rates of biological degradation of MT. Samples were pelleted, resuspended in Tris-HCl buffer 50 mm, pH 7.3 and sonicated (5 × 10 s) with an ultrasonic processor VC50 sonicator (Jencons, VWR, Lutterworth, UK). The protein content of the samples was estimated by the Bradford method (Bio-Rad). Rates of biological MT disappearance are expressed as nmol min^−1^ per mg protein and represent the average of three biological replicates.

For MTO *in vitro* assays, *R. pomeroyi* DSS-3 wild-type and *mtoX*^−^ were grown as above in the presence and absence of 0.5 mm MT for 6 h and pelleted. Cell pellets were washed three times with Tris-HCl buffer 50 mm, pH 7.3. Pellets were resuspended in 20 ml of Tris-HCl buffer and sonicated (as above). Cell lysates of 5 ml were placed in 20 ml serum vials to which 0.25 mm MT was added. MT concentration in the headspace was measured at time 0 and after 2 h of incubation at 28 °C by (GC) as described previously (Carrión *et al.*, 2015). Cell protein content and rates of biological degradation of MT were determined as described above.

There was no difference in the growth of the *mtoX* mutant strain compared to *R. pomeroyi* DSS-3 wild type in the presence of MT (0.5 mm).

### Transcriptional analysis of mtoX in *Ruegeria pomeroyi* DSS-3 and *Rhizobium leguminosarum*

The region of the *R. pomeroyi* DSS-3 genome that likely spanned the promoter of the SPOA0268-0272 operon was amplified from genomic DNA using primers GCGAATTCATCGAACCGCAATAGACCAC and GCCTGCAGGATCTTGGGCATATAGGGCG and cloned into the *lacZ*-reporter plasmid pBIO1878 (Todd *et al.*, 2012) to form an *mtoX-lacZ* fusion plasmids. The *mtoX-lacZ* fusion plasmid was digested with *Nsi*I and *Pst*I and religated to delete a ~800 bp 3′ fragment and form a SPOA0268-*lacZ* fusion plasmid. These plasmids were transferred by tri-parental conjugational mating (as above) into *R. pomeroyi* J470 and transconjugants were selected on rifampicin (20 μg ml^−1^) plus spectinomycin (200 μg ml^−1^). Transconjugants were grown overnight in MBM with succinate (10 mm) as carbon source (González *et al.*, 1997). The media either contained or lacked 5 mm DMSP, 1 mm MMPA, 0.1 mm MT or 0.1 mm DMS. The cells were assayed for β-galactosidase activity essentially as described previously (Rossen *et al.*, 1985).

### Identification of MTO homologs in bacterial genomes

MTO homologs were identified in microbial genomes based on BLASTP searches against assembled genomes at Integrated Microbial Genomes (IMG) (Markowitz *et al.*, 2009). MtoX amino acid sequences of *Hyphomicrobium* sp. VS, *R. pomeroyi* DSS-3 (locus SPOA0269) and *Methylophaga thiooxydans* (MDMS009_768) were used as queries. All hits used in further analysis had an *e*-value of 1e^−151^ or lower and a minimum pairwise identity at the level of the entire polypeptide of 52% or higher. On the basis of preliminary analyses showing support for a signal peptide in MTO, the start codons of two orthologous genes that appeared truncated were corrected to start at alternative start codons further upstream (locus GPB2148_3671 in marine gammaproteobacterium HTCC2148 was extended by 26 amino acids, while MDMS009_211 in *M. thiooxydans* was extended by 46 amino acids) as they appeared to have incomplete N-termini. Orthologs from *Phaeobacter* sp. LSS9 (714 amino acids) and *Comamonadaceae* bacterium EBPR_Bin_89 (335 amino acids) were excluded as the length of the polypeptides significantly deviated from the remaining range observed (410–491 amino acids). Sequences were aligned using CLUSTALW (Larkin *et al.*, 2007).

### Detection of *mtoX* homologs in metagenomic data sets

Metagenomic data sets were obtained from the CAMERA (Sun *et al.*, 2011) project website and searched for *mtoX* homologs using tblastn and the amino acid sequences of MtoX of *Hyphomicrobium* sp. VS, *M. thiooxydans* (locus tags MDMS009_211 and MDMS009_768) and *R. pomeroyi* DSS-3 (SPOA0269) as queries with a cutoff in *e*-value of 1e^−20^. In case of libraries that represented short read data (that is, <125 bp), a cutoff value of 1e^−05^ was used. Similarly, the metagenomic data sets were searched for homologs of the DMSP demethylase *dmdA* (*R. pomeroyi* locus SPO1913) and the bacterial housekeeping gene *recA* from *E. coli* at a cutoff of 1e^−20^ to estimate the fractional abundance of *mtoX-*containing cells in the bacterial community and compare it to that of the DMSP demethylase gene *dmdA*.

### Testing of MT oxidation in bacterial isolates

The potential to degrade MT by a range of pure cultures was assessed by monitoring changes in the MT concentration in the headspace after addition of 100 μM MT (Supplementary Table S4). Mineral salts medium was used to monitor MT oxidation without any other carbon source added. For *Methylococcus capsulatus* bath and *Methylocystis* sp., ATCC 49242 were tested for MT oxidation in NMS medium (Whittenbury *et al.*, 1970) that contained methane in addition to MT (20% v/v and 40% v/v methane added to the headspace for *M. capsulatus* and *Methylocystis*, respectively). *Pseudovibrio gallaeciensis* and *P. ascidiaceicola* were grown in marine broth (Difco) to which MT was added. Sterile controls were incubated for each medium used to account for chemical MT degradation.

### PCR amplification and cloning of MTO from enrichment cultures and environmental samples

PCR primers were designed based on an alignment of bacterial *mtoX* homologs (Supplementary Table S5). Primers were custom synthesized by Invitrogen Life Technologies (Paisley, UK) and initially tested using *Hyphomicrobium* sp. VS and *M. thiooxydans* DNA as template showing that a combination of primers 44F1/2 and 370R1/2/3 successfully amplified *mtoX* fragments from these two reference isolates. Further optimization of PCR conditions was carried out with DNA from additional bacterial isolates containing *mtoX* homologs and those showing potential for MT degradation (Supplementary Table S4). Unless noted otherwise, the PCR conditions used were 95 °C for 5 min, followed by 35 cycles of 95 °C for 1 min, 60 °C for 1 min, 72 °C for 1.5 min, followed by 72 °C for 5 min.

The presence and diversity of *mtoX* genes in enrichments and environmental samples was assessed using the newly designed primers on DNA extracted from *Brassica* rhizosphere soils enriched with dimethylsulfide or methanol (Eyice and Schäfer, 2016), DNA samples of ^13^C_2_-DMS stable isotope probing experiment carried out with soil samples (Eyice *et al.*, 2015), *Brassica oleracea* rhizosphere soil, and surface sediment from the river Dene (Wellesbourne, Warwickshire, UK). DNA was extracted from 2 ml of enrichment samples or 0.5 g of soil/sediment samples using the FastDNA Spin kit for Soil (MP Biomedicals, Santa Ana, CA, USA) according to the manufacturer’s instructions. In addition, *mtoX* diversity was assessed in surface sediments of a coastal saltmarsh (Stiffkey, Norfolk, UK). Five replicate sediment samples were obtained from the surface 5 mm oxic sediment layer of a small saline pool along transects starting at a patch of *Spartina anglica* plants at the periphery of the pool, extending 50 cm toward its center. The pH of the pool was 8.0, the water temperature was 16 °C. Samples were transported back to the laboratory on ice, before being centrifuged at 14 000 r.p.m. to remove the water and retain the sediment pellet. Samples were stored at −20 °C prior to DNA extraction. Extraction of DNA from the sediment samples was performed using a Qbiogene FastDNA SPIN Kit for soil (Thermofisher Scientific, Waltham, MA, USA), according to the manufacturer’s instructions. Eluted DNA was stored at −20 °C. PCR on Stiffkey sediment samples was carried out using primers MtoX41Fmodv2_inos and MTOX346Rmod (compare Supplementary Table S5) using a cycling regime consisting of a 95 °C hot start followed by 40 cycles of denaturation for 45 s, annealing for 45 s and elongation for 60 s at 95, 52 and 72 °C, respectively. A final extension step of 72 °C for 6 min followed. All PCR products were cloned in pCRTOPO 2.1 (Invitrogen Life Technologies). DNA sequencing of randomly chosen clones was carried out at the University of Warwick Genomics Centre using BigDye Terminator v3.1 cycle sequencing kit and ABI Prism 7900HT or ABI3100 sequence detection system (Applied Biosystems, Thermofisher Scientific, Waltham, MA, USA). Sequences of *mtoX* genes obtained from environmental samples have been deposited at the NCBI under accession numbers KY056824-KY057025.

## Results

### Purification and characterization of methanethiol oxidase from *Hyphomicrobium* sp. VS

We purified the native MTO enzyme from soluble extracts of *Hyphomicrobium* sp. VS grown on DMS or a combination of methanol and DMS using anion-exchange (MonoQ) chromatography followed by size-exclusion chromatography using a Superdex 75 column and another MonoQ column. Fractions exhibiting MTO activity and those adjacent on the final column run were analyzed on SDS-PAGE (Supplementary Figure S1A). From this it could be concluded that fraction 18 that exhibited MTO activity was dominated by a single polypeptide with an estimated molecular weight of 46 kDa. All other analyses were performed with this fraction. Electrospray-ionisation mass spectroscopy of this fraction revealed a polypeptide with a molecular mass of 46 186 Da (Supplementary Figure S2). Analysis of MTO by native gel electrophoresis suggested a molecular weight of ~180–200 kDa (Supplementary Figure S1B). Reanalysis of the excised band by SDS-PAGE resulted in a single band of 46 kDa (result not shown). Analytical gel filtration suggested an apparent size of 200 kDa (Supplementary Figure S1C) also indicating that MTO of *Hyphomicrobium* sp. VS is a homotetrameric enzyme.

The purified enzyme degraded MT and ethanethiol, but not methanol, methylamine or dimethylsulfide. When MT was the substrate, we found evidence for the production of formaldehyde, hydrogen sulfide and hydrogen peroxide, although we did not quantify the latter. The O_2_ dependency of MT conversion was shown by measuring activity with an oxygen sensor (Clark type). The ratio O_2_/MT consumed was around 0.75±0.05 (with 1.2 to 26 μm MT converted). This is lower than the 1.0 expected from the proposed stoichiometry (CH_3_SH+O_2_+H_2_O → HCOH+H_2_S+H_2_O_2_) and most likely caused by a very small contamination with highly active catalase reforming additional oxygen from hydrogen peroxide. This has been observed before (Suylen *et al.*, 1987). The remaining slow oxygen consumption after MT was depleted (rate dropping from 1.8 μm O_2_ per min to 0.2 μm O_2_ per min after 5 μm MT was depleted) was attributed to sulfide oxidation. Apart from being a (competitive) substrate for MTO, the sulfide produced in the MT oxidation was shown to be an inhibitor. This effect was also demonstrated before for the MTO purified from *Hyphomicrobium* strain EG (Suylen *et al.*, 1987). Adding Zn ions to the assay buffer to trap the produced sulfide resulted in 20% faster initial MT conversion rates (when tested at 5 μm MT, oxygen respiration increased from 5.5 to 6.7 μm O_2_ per min) and completely abolished the sulfide oxidation. Upon acidification of the final reaction mixture, at least 75% of the added MT sulfur was recovered as hydrogen sulfide. After including Zn^2+^ in the assay mixture, O_2_ consumption rates were constant (zero order kinetics) over almost the whole MT concentration range tested (1–20 μm MT). From this, it can be concluded that the *K*_m_ value is below 1 μm MT, which is below the detection limit of the respiration measurements. Using gas chromatographic analysis of MT (detection limit 0.05 μm MT), MT consumption rates at much lower concentrations could be tested. This resulted in a very low affinity constant (*K*_m_) for MT of 0.2–0.3 μm. The *K*_m_ for MT of the MTO was at least 10 × lower than previously reported values for *Hyphomicrobium* sp. VS (5–10 μm) and *T. thioparus* (31 μm) (Gould and Kanagawa, 1992; Pol *et al.*, 1994). This may be explained by the trapping of sulfide in our assays. The *V*_max_ was about 16 μmol mg^−1^ protein per min (Supplementary Figure S3). Formaldehyde was formed stoichiometrically, we observed formation of 4.1 nmol (±0.5) from 4 nmol of MT and 36.4 nmol (±2.6) from 40 nmol MT.

### MTO of *Hyphomicrobium* sp. VS is a metalloenzyme and Cu is involved in the redox process of MT oxidation

Inductively coupled plasma mass spectrometry showed that the purified enzyme preparation contained 3.5 mol Ca and 1.4 mol of Cu per mol of MTO tetramer (Supplementary Table S6). To further assess the potential role of Cu and Ca for MTO activity, we carried out chelation experiments using ethylenediamine tetraacetic acid (EDTA) and ethylene glycol tetraacetic acid (EGTA). Incubation of the enzyme with EDTA but not EGTA reduced the activity of MTO by 44% suggesting that Cu but not calcium has a role in the catalytic activity of MTO (Table 1).

A role of Cu in enzyme function was also supported by EPR spectroscopy and EXAFS for which a detailed description of the results is provided in the Supplementary Data. In brief, the EPR signals of resting and oxidized MTO samples did not have well-resolved signals that would be expected from Cu(II) mono-nuclear Cu site(s) (Supplementary Figure S4). Instead, there were signals that were probably due to two magnetically interacting Cu(II) centers, similar to Cu_A_ in cytochrome *c* oxidase or nitrous-oxide reductase, which are both binuclear copper centers, as well as Cu model complex possibly also without bridging sulfur (Antholine *et al.*, 1992; Solomon *et al.*, 1996; Monzani *et al.*, 1998; Kaim *et al.*, 2013). The changes in features in the EPR spectra with addition of substrate also indicated changes in the coordination of Cu when substrate binds (Supplementary Figure S5), which could indicate direct interaction of the substrate with the Cu center. Although at this point the exact nature of the Cu environment and status cannot be fully resolved, the data suggest that it is likely a binuclear site, as the data do not support a single atom Cu(II) center. Analysis of MTO by means of extended X-ray absorption fine structure (EXAFS) were consistent with the EPR data in that the oxidation state of the copper was between 1 and 2. The data indicated that the copper in the resting enzyme (in the absence of substrate) was coordinated by four nitrogen atoms with a Cu–N bond distance of 1.99 Å. EXAFS data from samples treated with substrate (methanethiol) or the reducing agent sodium dithionite showed that the copper was somewhat more reduced than the as-isolated, which was in line with an increased Cu–N bond length shown by the EXAFS data. The substrate-treated sample had fewer Cu–N ligands (2–3) than in the as-isolated enzyme. These observations are consistent with changes in oxidation state and coordination of the copper centers upon interaction with the substrate (Supplementary Figures S6 and S7) and support a role of Cu in the function of the enzyme.

### The identification of the gene encoding MTO reveals that MTO is a homolog of the selenium-binding protein family (pfam SBP56), has a conserved genomic context and that MTO is a periplasmic enzyme

The gene encoding MTO was identified based on N-terminal and *de novo* peptide sequencing against a draft genome sequence of *Hyphomicrobium* sp. VS. N-terminal sequencing of the purified MTO resulted in the identification of 15 amino acids, DETXNSPFTTALITG, with position X potentially a cysteine residue, indicating a processed N-terminus. In addition to the N-terminal sequence, internal peptide sequences were obtained (Supplementary Figure S8). Using peptide data in BLAST searches against the draft genome of *Hyphomicrobium* sp. VS available on microscope (Vallenet *et al.*, 2013), we identified the gene encoding MTO designated hereafter as *mtoX* (locus tag HypVSv1_1800007). A contig of 18.4 kb was assembled and confirmed by PCR and sequencing that contained a genomic region including the *mtoX* and additional genes downstream that are likely to be involved in its maturation (Genbank accession number KY242492). The *mtoX* gene is 1308 bp in size, encoding a polypeptide of 435 amino acids. Signal-P analysis (Bendtsen *et al.*, 2004) indicated that MTO contained a signal peptide with a predicted cleavage site at position 24 resulting in an N-terminus identical to the one determined experimentally of the purified MTO polypeptide. No transmembrane helices were identified by the software TMHMM (Krogh *et al.*, 2001) in the sequence representing the processed polypeptide, suggesting MTO to be a soluble periplasmic enzyme. The calculated molecular weight of the processed periplasmic MTO was 45 905 Da (46 192 Da assuming 4 Ca and 2 Cu in addition), in good agreement with the observed molecular weight on SDS-PAGE and the molecular weight estimated by Electrospray-ionisation mass spectroscopy (46 186). A conserved domain search with the predicted MTO amino acid sequence confirmed its homology to members of pfam05694, the SBP56 superfamily. A BLASTP search with the MTO protein sequence revealed hits with high homology in all three domains of life; including against bacteria (50–79% identity), archaea (26–29% identity) and eukarya (human SELENBP1 26% identity). The highest identities (77–79%) were with proteins annotated as selenium-binding proteins from other *Hyphomicrobium* species. Despite this similarity to known Se-binding proteins, no Se was found as judged by ICP elemental analysis. However, there are many cases of metalloproteins in which members of the same polypeptide family contain different specific metal co-factors, for example, in proteins of the FUR regulator superfamily (Fillat, 2014).

**Figure 2:**
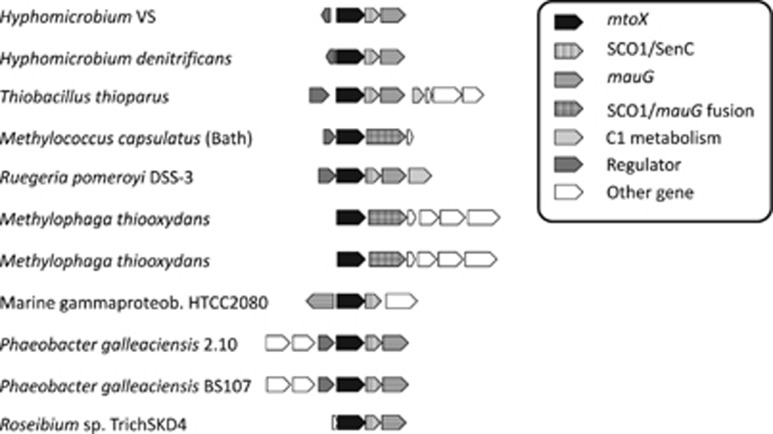
Genomic context of *mtoX* genes in selected bacteria showing the clustering of *mtoX* with genes encoding proteins containing SCO1/SenC and/or MauG domains, see inset for definition of coloring and patterns to particular gene annotation. As discussed in the text, in some instances, genes are encoding fusion proteins of SCO1 and *mauG* domains. Further information about the presence of SCO1 and MauG domain encoding genes in the vicinity of *mtoX* genes is given in Supplementary Table S7.

Genes downstream of *mtoX* in *Hyphomicrobium* sp. VS are predicted to encode homologs of the copper chaperone SCO1/SenC (Interpro: IPR003782) and of MauG, a protein with sequence similarity to diheme cytochrome *c* peroxidases that are required for the synthesis of tryptophan tryptophylquinone (TTQ) prosthetic groups (Wang *et al.*, 2003). The MTO, SCO1/SenC and MauG-encoding genes formed an operon-like structure (Figure 2). Based on the Cu content of MTO, the SCO1/SenC domain protein may be involved in MTO maturation. In *Paracoccus denitrificans*, the *mauG* gene encodes an enzyme responsible for post-translational modification of the methylamine dehydrogenase pre-protein to produce a protein-derived TTQ co-factor (Wang *et al.*, 2003).

### Phylogeny and distribution of *mtoX* in bacterial genomes

Homologs of *mtoX* were identified by BLASTX searches and a phylogenetic analysis was carried out based on the alignment of predicted amino acid sequences. This showed that MTO from *Hyphomicrobium* sp. VS belongs to a clade annotated as selenium-binding proteins (Supplementary Figure S9). In addition, the cluster with *Hyphomicrobium* sp. VS-MTO-like SBP included many organisms known to degrade one-carbon compounds (including DMS and MT), DMSP (for example, the model bacterium *R. pomeroyi* DSS-3) or sulfur-oxidizing bacteria. In many of these organisms, *mtoX* was also co-located with the *SCO1/senC* and *mauG* genes, or with genes encoding these two protein domains fused in a single gene as, for instance, in *M. thiooxydans* (Figure 2, Supplementary Table S7). In this marine gammaproteobacterium that degrades DMS via MT (Boden *et al.*, 2010), expression of polypeptides identified as selenium-binding protein was demonstrated during growth on DMS by peptide sequencing (Schäfer, 2007).

The capacity to degrade MT was tested in selected isolates. All tested bacterial strains containing the *mtoX* gene could degrade MT supporting a role for MTO in MT oxidation in these bacteria including *R. pomeroyi* DSS-3 (see below), *Hyphomicrobium denitrificans* (DSM1869), *M. capsulatus* (Bath), *Methylocystis* sp. ATCC 49242, *M. thiooxydans* DMS010, *T. thioparus* TK-m, *T. thioparus* E6, *Phaeobacter galleciensis* (DSM 17395) and *Pseudovibrio ascidiaceicola* (DSM 16392). Complete degradation of MT was observed within 2 days and was compared to sterile controls in which MT was not degraded over the same time period. In comparison, several strains that lacked the *mtoX* gene could not degrade MT, for example, *Methylophaga marina* and *Methylobacterium extorquens* AM1 (Supplementary Table S4).

### Genetic analysis of *mtoX* in *Ruegeria pomeroyi* DSS-3

*R. pomeroyi* DSS-3 produces MT as an intermediate, while catabolizing DMSP via the demethylation pathway (Reisch *et al.*, 2011b). The *R. pomeroyi mtoX* gene (SPOA0269), located on a megaplasmid, encodes a protein with 57% and 71% identity and similarity, respectively, to the MTO of *Hyphomicrobium* VS. To study the role of *mtoX* in MT degradation, SPOA0269 was replaced with a spectinomycin cassette in the *R. pomeroyi* genome.

MT removal assays conducted at the whole-cell level (0.5 mm MT) showed that wild-type *R. pomeroyi* had a rate of MT removal (23±1 nmol MT min^−1^ per mg protein) ~14-fold higher than those observed for *mtoX*^−^ mutant cultures (1.7±1.4 nmol MT min^−1^ per mg protein) supporting a role for MTO in MT oxidation (Table 2). The enzyme responsible for the low level MT removal activity remaining in the *mtoX*^−^ mutant was not identified.

Assays of MTO activity in cell lysates of wild-type and *mtoX*^−^ mutants with or without prior incubation with MT (0.5 mm) further support the role of the *mtoX* gene in MT oxidation and showed its activity to be inducible. Cell lysates of wild-type *R. pomeroyi* that had not been pre-incubated with MT consumed MT (0.25 mm) at a rate of 39±11 nmol MT min^−1^ per mg protein. In wild-type cultures pre-incubated with MT, the degradation rate increased fourfold to 139±26 nmol MT min^−1^ per mg protein. Cell lysates of *mtoX*^−^ mutants did not remove MT under the same conditions, irrespective of being pre-incubated in presence or absence of MT (Table 2). Thus, the *R. pomeroyi* gene SPOA0269 likely encodes a functional MTO enzyme whose level of MT oxidation was upregulated by exposure to MT.

### The transcription of *Ruegeria pomeroyi* DSS-3 *mtoX* is enhanced by MT

**Figure 3:**
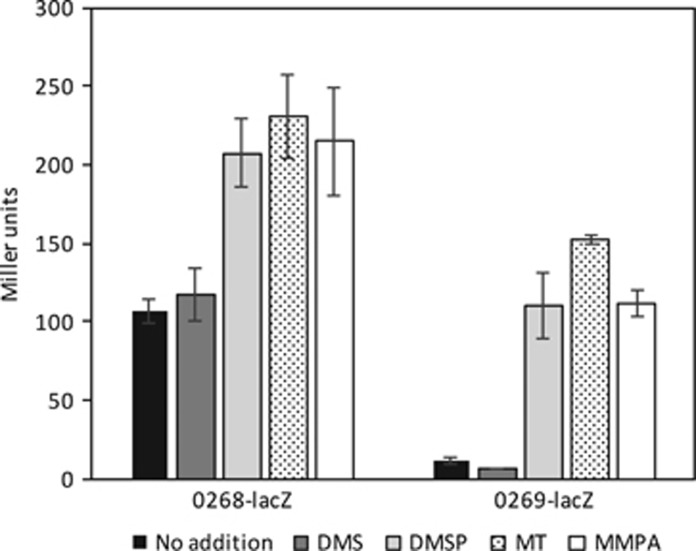
Transcriptional regulation of *Ruegeria pomeroyi* DSS-3 SPOA0268 and the methanethiol oxidase gene encoded by SPOA0269, assessed by beta galactosidase transcriptional fusion assay using various potential inducers. Values are reported in Miller units.

*R. pomeroyi* DSS-3 had a similar conserved *mtoX* gene neighborhood in which there is likely co-transcription with a gene encoding a SCO1/SenC domain protein (SPOA0270) and a *mauG*-like gene (SPOA0271) (Figure 2). Directly upstream of *mtoX* in *R. pomeroyi* is an IclR family transcriptional regulator (SPOA0268), and this gene arrangement is conserved in marine *Roseobacter* clade bacteria (Supplementary Table S7). We noted in microarrays carried out in (Todd *et al.*, 2012) that the transcription of the predicted operon (SPOA0268-0272) containing *mtoX* was significantly enhanced (two to fivefold) by growth of *R. pomeroyi* in the presence of DMSP. To confirm these observations, transcriptional *lac* fusions were made to the SPOA0268 and *mtoX* genes and assayed in *R. pomeroyi* in the presence of potential inducer molecules. Consistent with the microarray results, transcription of both SPOA0268 and *mtoX* was enhanced by DMSP, MMPA and most significantly by MT (~14-fold for *mtoX*), but not DMS (Figure 3). These results are consistent with the cell lysate assays and MT being the inducer molecule since both DMSP and MMPA are catabolized to MT by DMSP demethylation.

### Diversity of *mtoX* in environmental samples

The diversity of *mtoX* in environmental samples was assessed by PCR using newly designed primers 44F1/2 and 370R1/2/3 (Supplementary Table S5), which had been optimized by testing against a range of bacterial isolates. PCR with these primers resulted in amplicons of the expected size (~987 bp) (Supplementary Figure S10). Performing the PCR with DNA extracted from samples that were shown or would be expected to contain bacteria capable of methanethiol degradation (based on their known degradation of DMS and DMSP for instance) also yielded bands of the correct size. These samples included DNA extracted from DMS enrichment cultures from *Brassica* rhizosphere soil, bulk agricultural soil (Eyice and Schäfer, 2016), rhizosphere sediment of *S. anglica* (a DMSP-producing plant) obtained from Stiffkey saltmarsh (Norfolk, UK) and surface sediments of Stiffkey saltmarsh. Saltmarshes are known to be environments with high turnover of DMSP, DMS and MT (for example, Kiene, 1988a; Kiene and Capone, 1988b). Stiffkey saltmarsh samples used here had high DMS oxidation rates and enrichment of organisms containing *mtoX* genes was readily observed (Kröber and Schäfer; Pratscher *et al.*, unpublished data).

**Figure 4:**
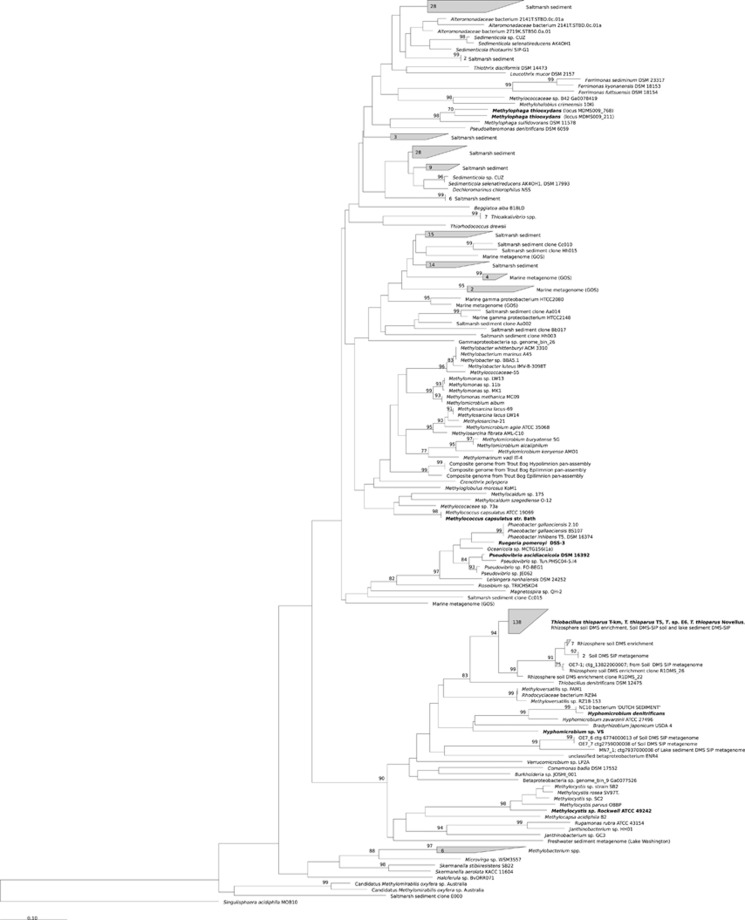
Phylogenetic analysis of translated methanethiol oxidase genes obtained from public databases, selected bacterial isolates by PCR, clone libraries of enrichment cultures and DNA extracted from surface sediments of Stiffkey saltmarsh. The tree was based on an alignment of full length and partial MtoX sequences in Arb and was derived using the neighbor joining algorithm and PAM correction implemented in Arb from a region comprising amino acid positions 85–300 of the *Hyphomicrobium* VS MtoX polypeptide. Bootstrap values (100 iterations) were derived in Mega 5, only those supporting terminal nodes with a confidence of 75% or higher are shown. Taxa shown in bold tested positive for MT oxidation.

Annealing temperatures used in these PCRs varied between 53 and 60 °C. The *mtoX* amplicons obtained with the *B. oleracea* rhizosphere DMS enrichment were cloned and clones chosen at random were sequenced. The *mtoX* gene sequences obtained belonged to two clades closely related to *T. thioparus* (Figure 4). Amplification efficiency of *mtoX* from DNA extracted from Stiffkey saltmarsh sediment samples was more variable and the primers were refined further (MtoX41Fmodv2_inos and MTOX346Rmod, Supplementary Table S5) to introduce degeneracies that improved their performance with these samples (result not shown). Surface sediment *mtoX* gene diversity was investigated in a tidal pool in Stiffkey saltmarsh using five independent samples from two transects across the pool. The analysis of randomly chosen clones from *mtoX* gene libraries prepared for these five surface sediment samples showed a high diversity of *mtoX* genes in the saltmarsh environment (Figure 4), while there appeared to be little variation in *mtoX* diversity between samples from the saltmarsh according to terminal restriction fragment length polymorphism analysis (result not shown). This showed Stiffkey saltmarsh *mtoX* sequences to belong to several distinct clades that lacked cultivated representatives. The *mtoX* sequences from Stiffkey saltmarsh clustered more closely with *mtoX* of gammaproteobacteria rather than those of alpha- or betaproteobacteria. The most closely related *mtoX* from cultivated strains were those of marine gammaproteobacterium HTCC2148, *Sedimenticola selenatireducens* and *Dechloromarinus chlorophilus*. MTO-encoding genes detected in DNA extracted from the DMS enrichments with *Brassica* rhizosphere soil and ^13^C-DNA of DMS-SIP experiments of soil and lake sediment samples (Eyice *et al.*, 2015) were related to betaproteobacterial taxa such as *T. thioparus* and *Methyloversatilis* sp. (Figure 4).

### Detection of *mtoX* in metagenomic data sets

Homologs of *mtoX* of *Hyphomicrobium* sp. VS, *M. thiooxydans* and *R. pomeroyi* were also detected in metagenomic data sets (Table 3). The relative abundance of *mtoX*-containing bacteria was estimated based on the frequency of detection of *mtoX* in comparison to *recA*, a universal housekeeping gene present in all bacteria and compared to that of *dmdA*, the DMSP demethylase. The relative abundance of *mtoX* varied across the different data sets (0–46%) and, in most cases, was lower than that of *dmdA*. On the basis of this analysis, it is difficult to delineate a general abundance pattern of *mtoX*-containing bacteria in different environments, however, it demonstrates that *mtoX* can be an abundant gene in some microbial communities. Selected *mtoX* sequences of sufficient length from the global ocean survey (Rusch *et al.*, 2007) and other metagenomic data sets were included in the phylogenetic analysis (Figure 4). Global ocean survey *mtoX* formed distinct clades, some of which were closely related to saltmarsh sediment *mtoX* types, or to the marine gammaproteobacterium HTCC2080, suggesting that most of the *mtoX* detected in metagenomics studies are originating from previously uncultured bacteria.

## Discussion

New insights into biochemical, genetic and environmental aspects of bacterial methanethiol oxidation presented here address a major knowledge gap in the biogeochemical sulfur cycle and the fundamental understanding of MT degradation by bacteria. Data presented here indicate that MTO is a periplasmic enzyme that is present in a wide range of bacteria, not limited to those known to produce MT as a metabolic intermediate during DMS and DMSP degradation, such as *Hyphomicrobium* VS, *Thiobacillus* sp. and *R. pomeroyi* DSS-3. The *mtoX* gene was also found in diverse cultivated bacteria that had not previously been recognized for their potential to degrade methanethiol. Homologous genes are also present in archaea and eukarya (including humans). In addition, the overall diversity of *mtoX* in environmental samples suggests that the potential for MT oxidation is also present in diverse uncultivated microorganisms and that MTO is a widely distributed enzyme in different terrestrial and marine environments, many of which have demonstrated potential for degradation of methylated sulfur compounds. MTO requires copper for its catalytic activity, and in *R. pomeroyi*, the gene encoding MTO is induced by MT. The enzyme from *Hyphomicrobium* sp. VS has a very high affinity for MT, with a *K*_m_ (0.2–0.3 μm) at least 10-fold lower than those previously reported, which may explain the low MT concentrations found in the environment.

Distinct molecular weights for MTOs from *Hyphomicrobium*, *Thiobacillus* and *Rhodococcus* strains have been reported previously. On the basis of high sequence homology of *mtoX* genes found in several *Hyphomicrobium* and *Thiobacillus* strains and the fact that previously purified MTOs from *Hyphomicrobium* sp. EG (Suylen *et al.*, 1987) and *T. thioparus* (Gould and Kanagawa, 1992) had similar molecular weights to the MTO of *Hyphomicrobium* sp. VS suggests that the previously purified MTOs are similar enzymes. Although previous studies reported MTO as a monomeric enzyme in *Hyphomicrobium* sp. EG and *T. thioparus* Tk-m (Suylen *et al.*, 1987; Gould and Kanagawa, 1992), rather than a homotetramer as in this study, these differences may be due to sensitivity of the MTO’s oligomeric state to pH. At pH 8.2, we found tetrameric MTO, but when we carried out analytical gel filtration at pH 7.2, as used by Suylen *et al.* (1987), MTO was detected in monomeric and tetrameric state (result not shown). Other observed differences between these MTOs may be due to different analytical approaches that were employed. For instance, a role of metals in MTO activity was previously ruled out based on chelation experiments, but these can fail to deplete the metals from the enzyme depending on variations in incubation conditions. The presence in and role of Cu for the functioning of the enzyme from *Hyphomicrobium* sp. VS is supported by ICP mass spectrometry analysis, changes in EPR spectra recorded with MTO in resting, reduced and oxidized state, and by chelation experiments showing a reduced activity of the enzyme. The presence of genes encoding putative Cu chaperones (SCO1/SenC) in close proximity to *mtoX* homologs in many bacterial genomes provides further circumstantial evidence for a role of copper in MT oxidation and provides a focus for future genetic and biochemical studies.

Besides the presence of a *mauG* homolog, involved in maturation of a protein-derived TTQ co-factor in methylamine dehydrogenase, we found supporting evidence that the MTO also contains a TTQ co-factor. The PDB database contains the structure of the heterologously expressed SBP56 protein of *Sulfolobus tokodaii* (PDB entry: 2ECE). Analysis of the structure of this non-matured protein (no copper, no TTQ) made it possible to identify the putative ligands involved in copper binding (histidines) and TTQ synthesis (tryptophans) in the *Sulfolobus* homolog (Supplementary Figure S11). Alignments of the tryptophan and histidine residues identified showed strict conservation over the three domains of life. EPR and EXAFS analyses suggest that Cu in MTO of *Hyphomicrobium* sp. VS is coordinated by four nitrogen atoms, which would fit with the strictly conserved histidine residues which in *Hyphomicrobium* sp. VS-MTO are His89, His90, His140, His412 (Supplementary Figures S8 and S12). The structural information and the presence of the *SCO1/senC* and *mauG*-like genes support the presence of a TTQ co-factor and two copper atoms per monomer; further, if we assume 4 Ca and 2 Cu per monomer, the calculated mass exactly fits the Electrospray-ionisation mass spectroscopy analysis: 46 193 vs 46 186 Da. The arrangement of the genes *mtoX*, *SCO1/senC* and *mauG* encoding MTO, a copper chaperone, and homolog of the enzyme known to be involved in maturation of a protein-derived TTQ co-factor in methylamine dehydrogenase was highly conserved in a wide range of bacteria (Figure 2 and Supplementary Table S7).

The role of MTO in metabolism of MT and DMSP as well as its transcriptional regulation were demonstrated in *R. pomeroyi* showing that this enzyme has an important role in metabolism of DMSP. Transcriptional fusions of the IclR type regulator upstream also demonstrated that MT as well as DMSP and MMPA (which are degraded to MT) induced MTO transcription. Interestingly, despite the presence of a functional MTO, it has long been known that *R. pomeroyi* DSS-3 liberates MT when grown in the presence of DMSP, this being one of the products of the DMSP demethylation pathway (Reisch *et al.*, 2011b). Thus, under these circumstances, the MTO does not have sufficient activity to oxidize all the DMSP-dependent MT that is formed. However, we noted (unpublished) that the *mtoX*^−^ mutant *R. pomeroyi* DSS-3 released more MT (~1.5-fold) when grown in the presence of DMSP than did the wild type.

The identification of the gene encoding MTO in bacteria has allowed assessing the distribution of the enzyme in the environment and identified its evolutionary relationship to the selenium-binding protein family (SBP56), a protein family that has as yet an unresolved function. Metal analysis by ICP mass spectrometry did not show the presence of selenium in MTO. SBP56 is a highly conserved intracellular protein (Bansal *et al.*, 1989). Previous reports stated that it is involved in the transport of selenium compounds, regulation of oxidation/reduction and late stages of intra-Golgi protein transport, but its exact role has remained unclear (Jamba *et al.*, 1997; Porat *et al.*, 2000; Ishida *et al.*, 2002). Homologs of SBP56 were found in human, mouse, fish, horse, birds, abalone and plants such as *Arabidopsis thaliana* and maize in addition to bacteria and archaea (Jamba *et al.*, 1997; Flemetakis *et al.*, 2002; Self *et al.*, 2004; Song *et al.*, 2006). The human SBP56 homolog has been shown to be a methanethiol oxidase ((Pol *et al.*), *Nat Genet*, in revision). To what extent the other SBP56 have similar function to MTO needs to be addressed, but a possible relationship of SBP56 with C1 metabolism was previously pointed out based on the presence of the SBP56-encoding gene in the vicinity of genes encoding selenocysteine-containing formate dehydrogenases in the genome of *Methanococcus vannielli* and *M. maripaludis* (Self *et al.*, 2004).

Homologs of *mtoX* are present in a wide range of bacteria, and metagenomes from marine pelagic, coastal, hydrothermal and terrestrial environments, including DMS stable isotope probing experiments of soil and lake sediment samples. On the basis of processes that contribute to MT production in marine and terrestrial environments, a wide distribution of this enzyme is not surprising. The diversity of *mtoX*-containing organisms present in the environment is currently not well represented by isolated organisms, which suggests that the ability to degrade MT is more widely distributed than currently realized. This lack of environmentally relevant model bacteria limits our ability to appreciate which organisms are important as sinks for MT in different environments, how the expression of MTO in these organisms is regulated and which other degradative capabilities they may have. Using a stable isotope probing approach with ^13^C_2_-DMS, we recently identified *Methylophilaeceae* and *Thiobacillus* spp. as DMS-degrading bacteria in soil and lake sediment (Eyice *et al.*, 2015). The finding of *mtoX* genes in representatives of *Thiobacillus* and *Methylophilaceae* is consistent with the role that MT has as a metabolic intermediate in previously characterized DMS-degrading bacteria such as *Thiobacillus* spp. and adds further weight to the suggestion that certain *Methylophilaceae* have the metabolic potential to degrade DMS. The detection of *mtoX* in a saltmarsh environment is in agreement with such environments being hotspots of organic sulfur cycling (Steudler and Peterson, 1984; Dacey *et al.*, 1987) based on production of DMSP and DMS by benthic microalgae, macrophytes and macroinvertebrates (Otte *et al.*, 2004; Van Alstyne and Puglisi, 2007), and MT production through anaerobic processes in the sediment (Lomans *et al.*, 2002).

Overall, this study adds to our fundamental understanding of a key step in the sulfur cycle. The identification of the gene encoding this enzyme reveals its homology to a protein superfamily of which homologs are present in organisms ranging from bacteria to humans, but for which only sketchy functional information has been reported previously. The outcomes of this study will therefore facilitate future investigations of the role of MTO homologs in a wide range of organisms by providing testable hypotheses regarding its physiological relevance in these organisms. At the same time, the identification of the gene encoding MTO as well as its metal dependence will provide key foci for investigation of the diversity and distribution of MTO and potential constraints on its activity such as metal availability on MT degradation rates in the environment as well as aspects of the catalytic mechanism of MTO.

## Figures and Tables

**Table 1:** Effect of chelators on the activity of *Hyphomicrobium* VS methanethiol oxidase

*Sample*	*Specific activity* *(μmol MT min*^−*1*^ *mg*^−*1*^ *protein)*
MTO—no chelator	12.9±1.5
MTO—EDTA-treated	5.7±1.1
MTO—EGTA-treated	12.5±0.9

Abbreviations: EGTA, ethylene glycol tetraacetic acid; MT, methanethiol; MTO, MT oxidase.

**Table 2:** MT consumption by whole cells and lysates of *R. pomeroyi* DSS-3 wild-type and mtoX^−^ strains (*n*=3)

*Sample*	*MT consumption (nmol MT min*^−*1*^* per mg protein*)
*Whole-cell assays with 0.5 mm MT*	
Wild type	23.4±1.2
*mto*X^−^	1.7±1.4
	
*Cell lysate assays with 0.25 mm MT from cells pre-incubated in the presence (0.5 mm, +MT) or absence of MT (−MT)*	
Wild-type −MT	39.2±14.4
Wild-type +MT	139.2±25.5
*mto*X^−^ −MT	No MT degradation
*mto*X^−^ +MT	No MT degradation

Abbreviation: MT, methanethiol.

MT consumption is expressed as nmol MT removed min^−1^ per mg protein.

**Table 3:** Analysis of metagenomic data sets for the presence of *mtoX*, *dmdA* and *recA* homologs

*Metagenome name (CAMERA project name)*	*Biome*	*No. of sequences*	*Number of hits*			*Estimate*		*See footnote*	*CAMERA/imicrobe/NCBI data set accession*
			*mtoX*	*dmdA*	*recA*	*% Of cells with mtoX*	*% Of cells with dmdA*		
Antarctica aquatic microbial metagenome	Antarctic lake	64 626 265	230	533	504	45.6	106		PRJNA33179
Botany bay metagenomes	Coastal marine pelagic	15 538 531	95	551	511	18.6	108		CAM_PROJ_BotanyBay
Western channel observatory microbial metagenomic study	Coastal marine pelagic	7 354 754	46	622	623	7.4	100		CAM_PROJ_WesternChannelOMM
Metagenomic analysis of the North Atlantic spring bloom	Marine pelagic	6 784 781	8	268	510	1.6	53		CAM_PROJ_BATS
Microbial community genomics at the HOT/ALOHA	Marine pelagic	5 687 251	10	524	534	1.9	98		CAM_PROJ_HOT
North Pacific metagenomes from Monterey Bay to Open ocean (CalCOFI line 67)	Marine pelagic	5 618 147	7	4	117	6.0	3		CAM_P_0000828
Monterey bay transect CN207 sampling sites	Coastal marine pelagic	5 248 980	19	230	514	3.7	45		CAM_P_0000719
Guaymas Basin deep-sea metagenome	Marine deep water	4 970 673	56	69	340	16.5	20		CAM_P_0000545
Marine metagenome from coastal waters project at Plymouth marine laboratory	Coastal marine pelagic	1 444 540	3	79	172	1.7	46		CAM_PROJ_PML
Marine bacterioplankton metagenomes	Marine pelagic	1 314 590	1	80	239	0.4	33		CAM_PROJ_Bacterioplankton
Sargasso sea bacterioplankton community	Marine pelagic	606 285	11	21	91	12.1	23	a	CAM_PROJ_SargassoSea
Sapelo island bacterioplankton metagenome	Coastal marine pelagic	354 908	9	14	30	30.0	47	b	CAM_PROJ_SapeloIsland
Washington lake metagenomes	Lacustrine	252 427	4	12	75	5.3	16		PRJNA30541
Two HOT fosmid end depth profiles (HOT179 and HOT186)	Marine pelagic	194 593	2	20	54	3.7	37		CAM_P_0000828
Waseca county farm soil Metagenome	Soil	139 340	1	4	16	6.3	25	c	CAM_PROJ_FarmSoil
Hydrothermal vent Metagenome	Marine hydrothermal vent	49 636	1	0	28	3.6	0		CAM_PROJ_HydrothermalVent

Abbreviation: DMSP, dimethylsulfoniopropionate.

a The distribution of hits against sampling sites (‘control’ or ‘DMSP’) in the Sargasso sea bacterioplankton study was as follows: *mtoX* 7 control, 4 DMSP; *dmdA* 4 control, 17 DMSP; *recA* 42 in control, 49 in DMSP.

b Because of the very short reads in Sapelo Island bacterioplankton metagenome an *e*-value cutoff of 1e^−05^ was used. Hits at that level had a high pairwise similarity, for *dmdA*, there were shorter 100% identity hits with higher *e*-values than the cutoff used, which were therefore rejected by this approach suggesting this as a stringent cutoff value.

c The *dmdA* hits in the Waseca county farm soil study had low maximum pairwise identities between 24 and 29% at the amino acid level.
