# Supplementary material for: Bacterial SBP56 identified as a Cu-dependent methanethiol oxidase widely distributed in the biosphere
Source: ISME J. 2017 Oct 24;12(1):145–60. doi: 10.1038/ismej.2017.148 (PMC5739008; doi:10.1038/ismej.2017.148)
Supplement: Supplementary Information [file ismej2017148x1.pdf]

Bacterial SBP56 identified as a Cu-dependent methanethiol oxidase widely distributed in the biosphere

Özge Eyice, Nataliia Myronova, Arjan Pol, Ornella Carrión, Jonathan Todd, Tom J. Smith, Stephen J. Gurman, Adam Cuthbertson, Sophie Mazard, Monique A.S.H. Mennink-Kersten, Timothy D.H. Bugg, K. Kristoffer Andersson, Andrew W.B. Johnston, Huub J.M. Op den Camp, Hendrik Schäfer

Supplementary Information

This document contains supplementary information including additional detail concerning methods and results of methanethiol oxidase protein purification and characterization, Electron Paramagnetic Resonance Spectroscopy (EPR) analysis, X-ray spectroscopy analyses (EXAFS), genome sequencing of *Hyphomicrobium* sp. VS (Supplementary Figures S1-S12, Supplementary Tables S1-S7).

Protein purification

An initial protocol for purification of methanethiol oxidase from *Hyphomicrobium* sp. VS was developed as shown below in Supplementary Table S1. This resulted in a protein preparation that revealed a single dominant polypeptide in SDS-PAGE analysis with a molecular weight of approximately 46 kDa (Kertens-Mennink, Pol, Op den Camp, unpublished results). The activity of methanethiol oxidase in this preparation was enriched 36-fold in this fraction.

Supplementary Table S1. Purification Table for preliminary MTO purification.

	volume	MTO activity (µmol MT/min)	Protein [mg]	Purification fold	Recovery
crude extract	5.0	4.8	135.0	1.0	100.0
Q-sepharose	7.4	3.5	12.7	7.8	73.3
Amicon filtration	2.0	4.1	10.3	10.9	84.0
Superose 12	12.0	4.7	3.3	40.2	98.0
Amicon filtration	6.1	1.8	3.9	12.8	36.7
TSK-DEAE	7.0	0.5	0.4	36.0	10.8

Notes: after the second Amicon filtration, only 26.3% of the material was purified further, leading to a recovery of 10.8%. The theoretical recovery would therefore be 40% if all material had been further purified.

The above purification procedure (Supplementary Table S1) resulted in an active enzyme preparation showing a single dominant polypeptide on SDS-PAGE of a molecular weight of ~45-46 kDa (result not shown). The final purification protocol adopted for MTO was as described in Materials and Methods of the main text, consisted of sequential steps of anion exchange chromatography on a MonoQ 10/100 column followed by size exclusion chromatography using a Superdex-75 column. This also resulted in a protein preparation that was dominated by a 46 kDa polypeptide. The fraction with MTO activity eluting from the anion-chromatography run again showed a single dominant polypeptide of 46 kDa, which was up to 50-fold enriched in specific activity in neighbouring fractions compared to the cleared supernatant (Supplementary Table S2). This demonstrated that the enzyme activity responsible for MT degradation in *Hyphomicrobium* sp. VS was successfully enriched.

Supplementary Table S2. Modified purification scheme, comparison of specific enzyme activity in active fraction recovered after MonoQ anion exchange chromatography with initial soluble enzyme extract after removal of cell membranes

	protein in assay [mg]	MT degraded [µmol] in 30 min	specific activity (µmol/min mg protein)	fold-purification
Supernatant	1.56	10	0.21	1.0
MonoQ fraction 18	0.17	54	10.58	50.4

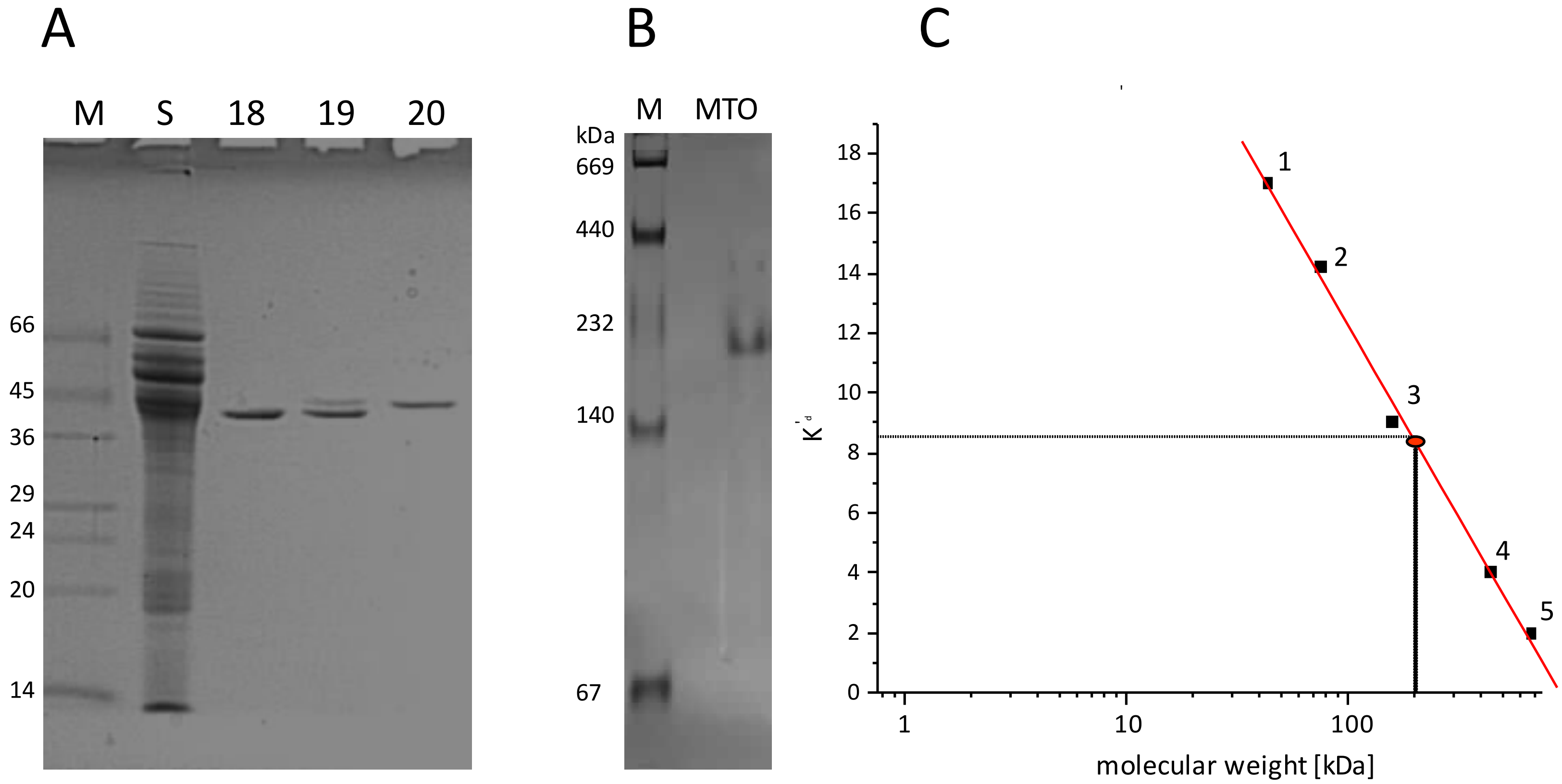

Supplementary Figure S1. (A) SDS-PAGE analysis of protein fractions of *Hyphomicrobium* sp. VS. M, molecular weight marker (weights in kDa); S, soluble fraction after removal of membrane fraction; 18, 19 and 20 are fraction numbers of the Mono-Q run. **(B)** Native (10% w/v polyacrylamide) gel electrophoresis analysis of MTO purified from *Hyphomicrobium* VS. Lanes: MTO, methanethiol oxidase; lane M, molecular size standards (Mw given in kDa). **(C)** Calibration plot of the analytical gel filtration showing for estimation of the molecular weight of MTO. Standards used for column calibration: 1, ovalbumin (43 kDa); 2, conalbumin (75 kDa); 3, aldolase (158 kDa); 4, ferritin (440 kDa); 5, thyroglobulin (669 kDa), the relative elution of MTO is indicated giving an estimated Mw of approx 200 kDa.

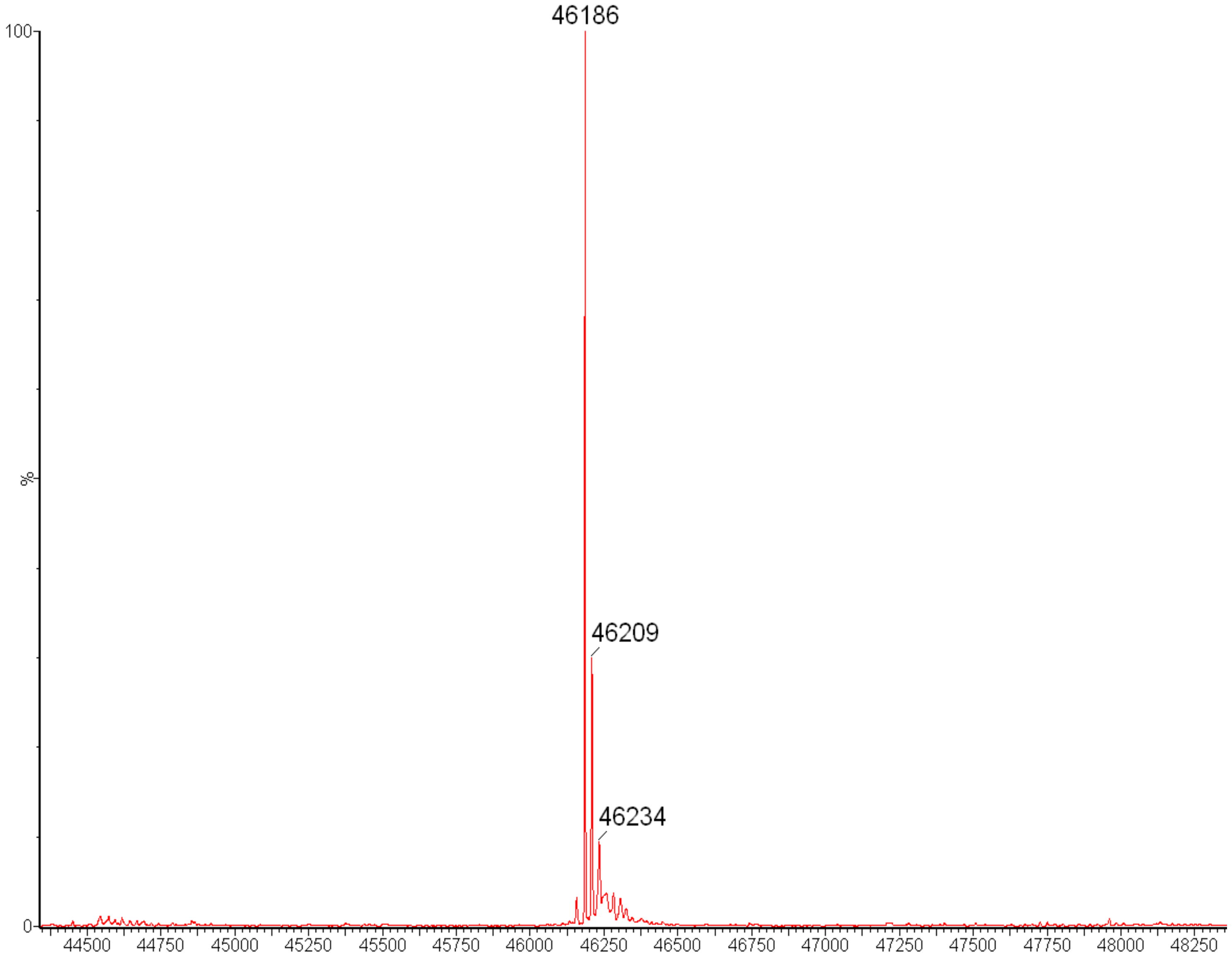

Supplementary Figure S2. Electrospray ionisation mass spectrometry analysis of purified MTO shows molecular weight of the subunit to be approximately 46.2 kDa.

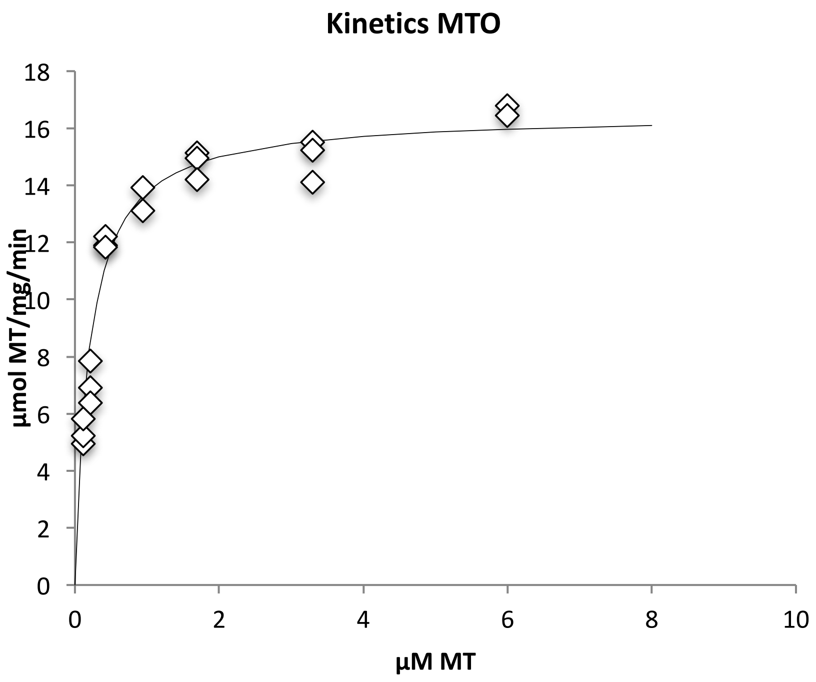

Supplementary Figure S3. Kinetic analysis of methanethiol oxidase from *Hyphomicrobium* sp. VS. Based on the data shown in the plot, the estimated K_m for MT was 0.2-0.3μM and the V_{max} was approximately 16μmol mg^{-1} protein min^{-1}.

Electron Paramagnetic Resonance Spectroscopy – Results

In resting and oxidized samples we observed EPR signals at temperatures of 7 and 13 K (Supplementary Fig S4). The resting oxidized MTO EPR signals did not have well-resolved Cu(II) EPR signals from an isolated mono-nuclear Cu(II) site(s), suggesting that the Cu-ions are coupled/interacting between Cu ions and/or with a radical in some redox state (Supplementary Fig S4). We could observe trace amounts of a radical with a very narrow signal centered around 2.0 (as it is a first derivative of a peak it has both positive and negative features). These broad signals were probably from two magnetically-interacting Cu(II) centres, with positive signals before 2.05, somewhat similar to Cu_A in cytochrome *c* oxidase or nitrous-oxide reductase, both binuclear copper centres, as well as Cu model complex possibly also without bridging sulfur (Antholine et al 1992, Monzani et al 1998, Solomon et al 1996, Kaim et al, 2013). These could be species from a Cu(II)Cu(I)/ Cu(1.5)Cu(1.5) cluster. The oxidized samples also had a signal around g=4.8 (see Supplementary Fig S5 at 7 and 13 K) indicating spin-coupled or spin-interacting species at this high g-value. The EPR spectra (Supplementary Figures S4 and S5) have several features of a Cu protein that cannot come from a single Cu site as the positive signals at g = 2.46 lack other resolved hyperfine interactions from a single isolated Cu (it is also a very weak Cu(II) at higher g-values visible with 4 hyperfine couplings resolved). The g= 2.39 and 2.24 could be linked to the 2.46 signal but all these three features did not look like normal hyperfines but could possibly indicate that sulfur may be a ligand. The g=2.053 is a zero crossing signal (like the radical) with both positive and negative signal and might be linked to any of the 2.46, 2.39 or 2.24 signals, but at this point of time it is unclear which. The last negative feature with g= 1.905 has unusually low g-value that might be seen in spin-interacting signals (Andersson et al 2003).

In summary, the changes in features in the EPR spectra indicate changes in coordination of Cu when substrate binds, which could indicate direct interaction of the substrate with the Cu centre. Although at this point the exact nature of the Cu environment and status cannot be fully resolved, it is likely to be a binuclear site, as the data do not support a single atom Cu centre.

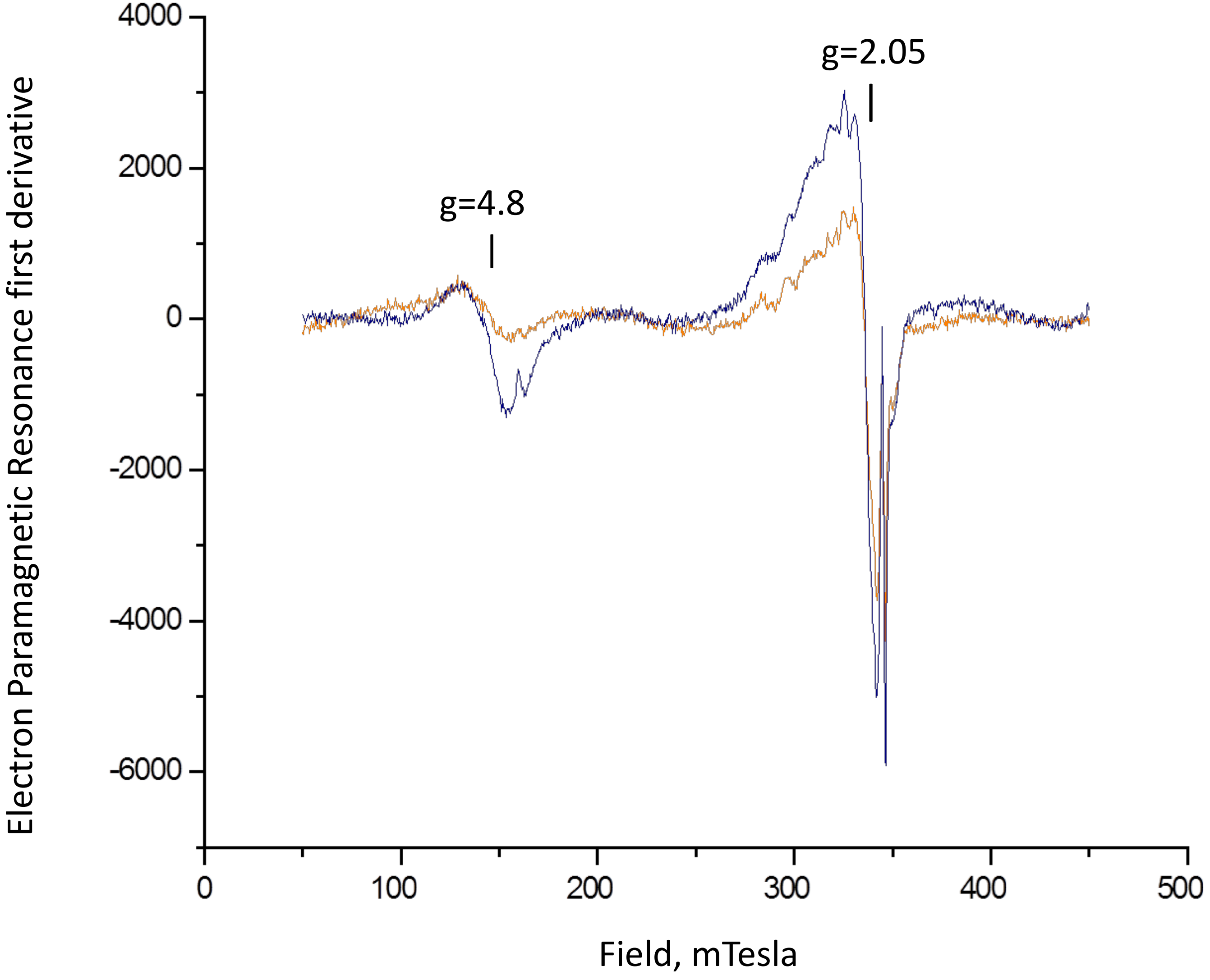

Supplementary Figure S4. Oxidized MTO at 7 K (blue) and 13 K (orange). MTO 9.2 mg/ml, 10 mM TRICINE, pH 8.2, 1 mM benzamidine, 1.8 mM sodium hexachloroiridate (V).

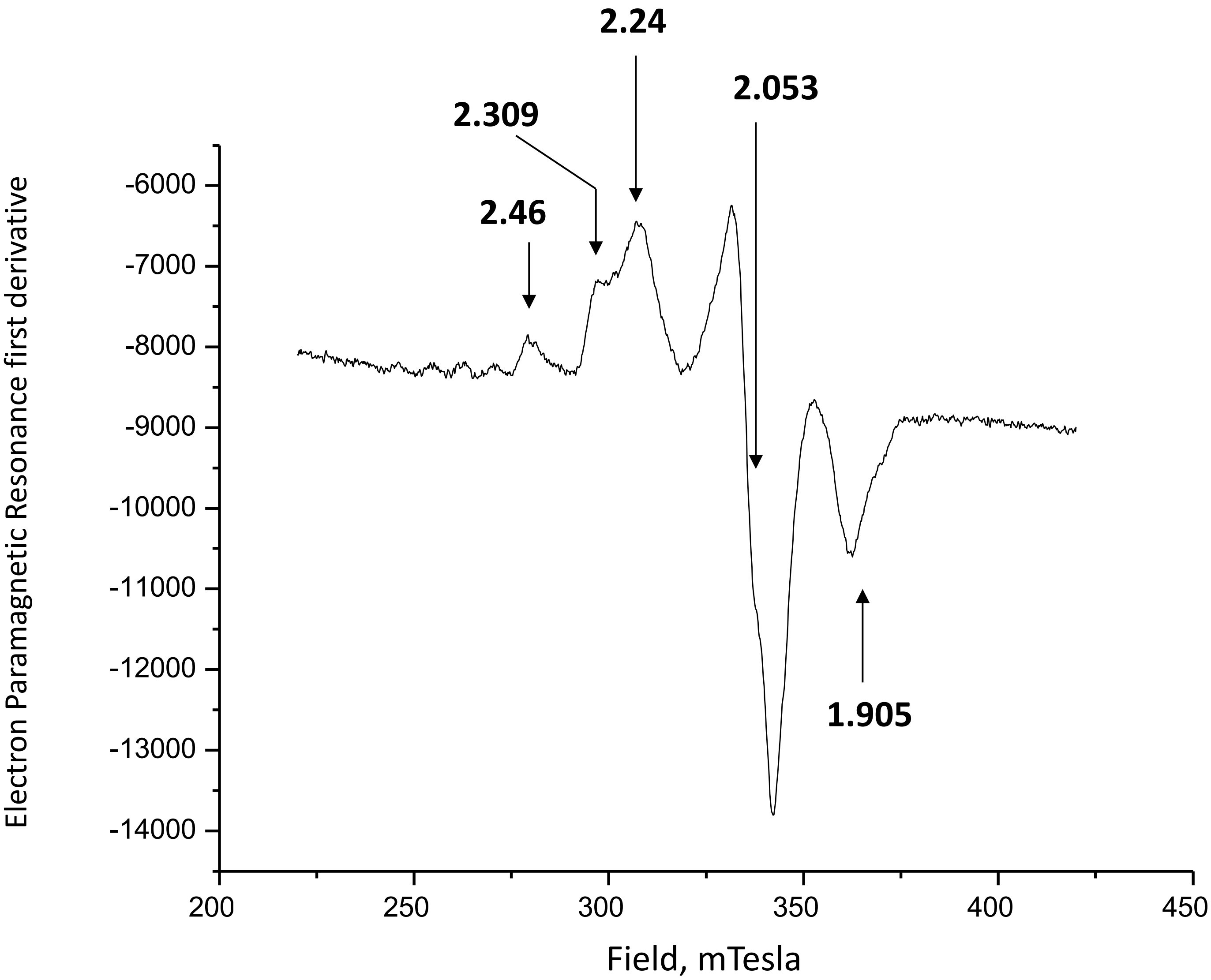

Supplementary Figure S5. Electron Paramagnetic Resonance Spectroscopy analysis of oxidized MTO (MTO with ethanethiol and oxygen) at 15 K and microwave power 200 µW.

X-ray Spectroscopy Analysis of Methanethiol Oxidase

Detailed Methods

X-ray absorption spectra were obtained in fluorescent mode on station B18 of the Diamond Light Source (Didcot, UK). This uses the technique of quick EXAFS (QuEXAFS), where the monochromator rotates at a constant rate during data acquisition. The fluorescence was detected using a nine element germanium solid state detector. Data were obtained at the Cu K edge for a variety of samples and standards. All data were obtained with the samples at 77K in a cryostat. To minimize radiation damage the beam was rastered across the sample, which was moved between each scan. Each scan took about 20 minutes to acquire.

The fluorescence signals were merged and normalized using the Athena program. The output from this program is used in studies of the absorption edge shape and the chemical shifts. The background was then subtracted using the Pyspline program to produce the oscillatory EXAFS spectrum. This was analysed using the EXCURV program. This employs the rapid curved wave theory, including multiple scattering when necessary (Gurman et al. 1986). Scattering properties of the atoms were calculated *ab initio* within the program. The structure surrounding the Cu atom is described in terms of shells of atoms: a shell is a set of atoms of the same chemical type at the same average distance. The structural parameters fitted are the number and type of atom within a shell, their average distance from the Cu atom and the mean square deviation in this distance (the Debye-Waller factor).

Copper metal (foil), CuO and CuS were used as reference samples. The copper-containing enzyme tyrosinase (Sigma Aldrich, Gillingham, UK) was used as additional reference. Five samples of purified MTO were analysed: as-isolated enzyme (MTO1 and MTO2); enzyme treated with the oxidising agent sodium hexachloroiridate (2 mM) (MTO3); enzyme treated with the substrate methanethiol (MTO4); enzyme treated with the reducing agent sodium dithionite (1 mM) (MTO5).

In fitting the EXAFS from the tyrosinase standard sample it became apparent that the spectrum was contaminated by a copper metal signal. This probably arose from fluorescence from the cryostat window mounts which are hit by scattered primary X-rays, and will also occur in all other samples. Fitting the EXAFS spectrum enabled us to determine the proportion of copper metal signal in the data. This was then subtracted from the fluorescence data to obtain corrected spectra for use in the analysis. The nearest neighbour (Cu-N) structural data obtained from the EXAFS fits were the same in the original and corrected data. However, the presence of this contaminating signal means that it is not possible to determine whether there is any Cu-S coordination in any of the samples (Cu-Cu at 2.51 Å, Cu-S at about 2.65 Å). Edge spectra corrected in this way were used for the edge analysis.

In studies of the edge structure the main interest is in the energy of the edge, which gives the chemical shift. The position of the edge is usually defined by the half way point (E_{50}). This could not be used here this because of the structure on the edge, which was interpreted as arising from transitions to the unoccupied Cu 4p level. Instead, the 70% absorption point (E_{70}) was used as a proxy for the position of the ionization threshold and the 30% absorption point (E_{30}) as a proxy for the energy of the 3p level. Increasing ionization of the copper atom will increase the energy of the ionization threshold and decrease the energy of the 4p level relative to this because of changes in electron screening.

EXAFS Results

EXAFS analysis (Figure S6) of the as-isolated MTO (samples MTO 1 and 2) and the hexachloroiridate-oxidised samples gave very similar data. These data show Cu coordinated by four light atoms (which are presumed to be nitrogen based upon the availability of histidines as ligands) with a 1.99 Å Cu-N distance. This bond distance is comparable to a three coordinate Cu-N bond distance of 1.94 Å obtained from a sample of the model copper enzyme tyrosinase that was analysed via EXAFS in parallel with the MTO samples, and a Cu-N distance of 1.97-2.05 Å in the X-ray crystal structure of peptidylglycine α-hydroxylating monooxygenase (Chauhan et al., 2014; PDB accession number 1YI9) and in the complex between copper and the peptide Gly-Leu-Tyr moiety with a 4 N square pyramid and a Cu-N distance of 1.92 Å (van der Helm and Franks, 1968). The hexachloroiridate treated sample (MTO 3) showed the presence of three nitrogen ligands at 1.98 Å and has at least 1-2 light atom ligands (N or O).

The sample treated with the substrate (methanethiol) (MTO 4) showed 2-3 ligands at a Cu-N bond distance of 2.04 Å. The dithionite-reduced sample (MTO 5) gave structural data that were somewhat unclear, with about four nitrogen ligands at a Cu-N bond distance of about 2.05 Å.

The position and shapes of the Cu-K absorption edge suggest that the oxidation state of the Cu in the as-isolated (MTO 1 and 2) and hexachloroiridate-treated (MTO 3) samples is between 1 and 2. The samples treated with substrate (MTO 4) and sodium dithionite (MTO 5) are somewhat more reduced than the other samples. This conclusion is in line with the increased Cu-N bond length in the last two samples (and illustrated by the correlation shown in Figure S7). The results are also consistent with a redox enzyme in which copper undergoes changes in coordination and oxidation state during turnover. Whilst the corrected data do not give any information about whether the substrate sulphur atom becomes ligated to the copper, they do indicate a reduction in the number of light-atom (presumably nitrogen) ligands and a decrease in the oxidation state of copper upon binding of the substrate.

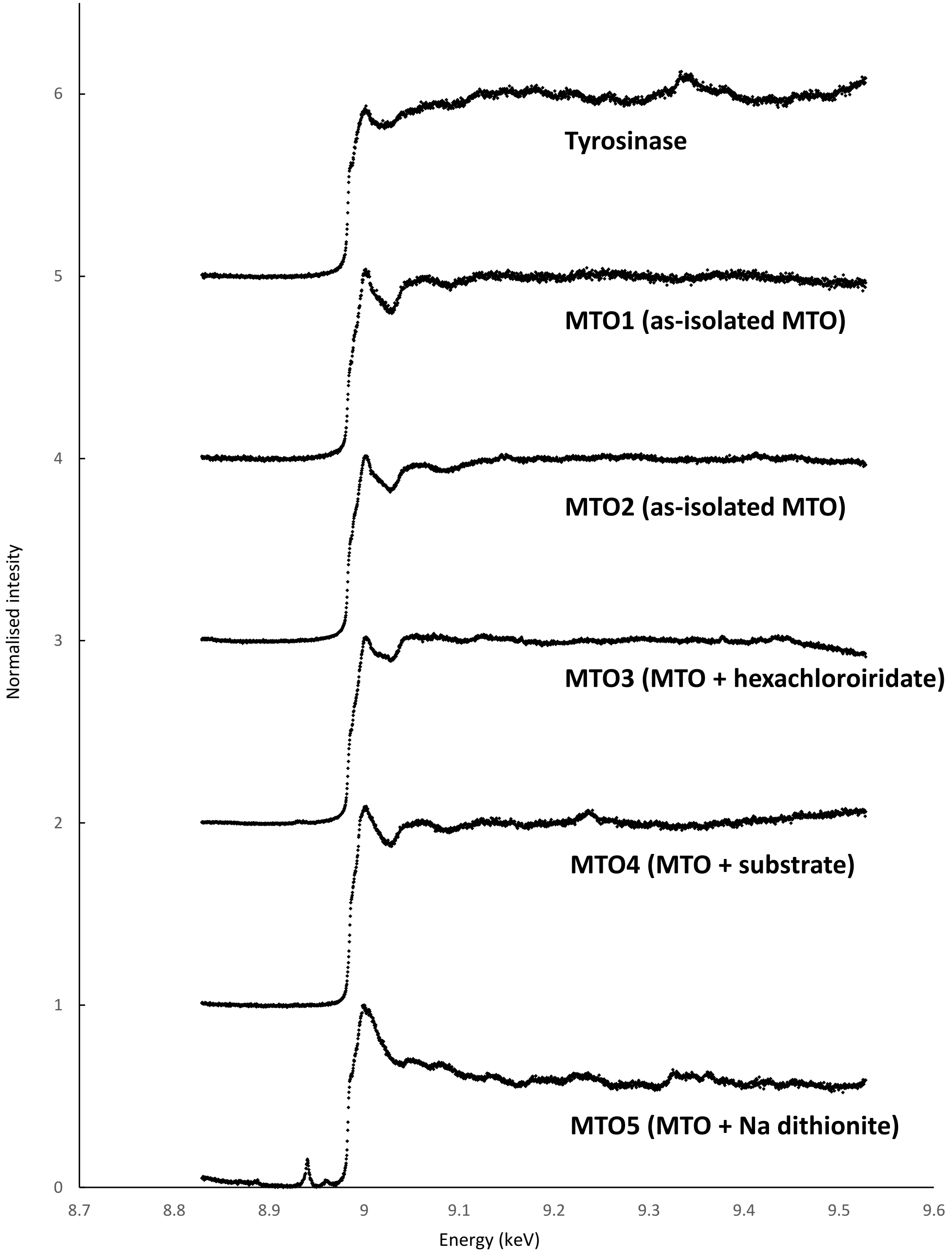

Supplementary Figure S6. Normalised X-ray spectra of the MTO and tyrosinase samples, corrected to remove the signal due to copper metal contamination as detailed in the text.

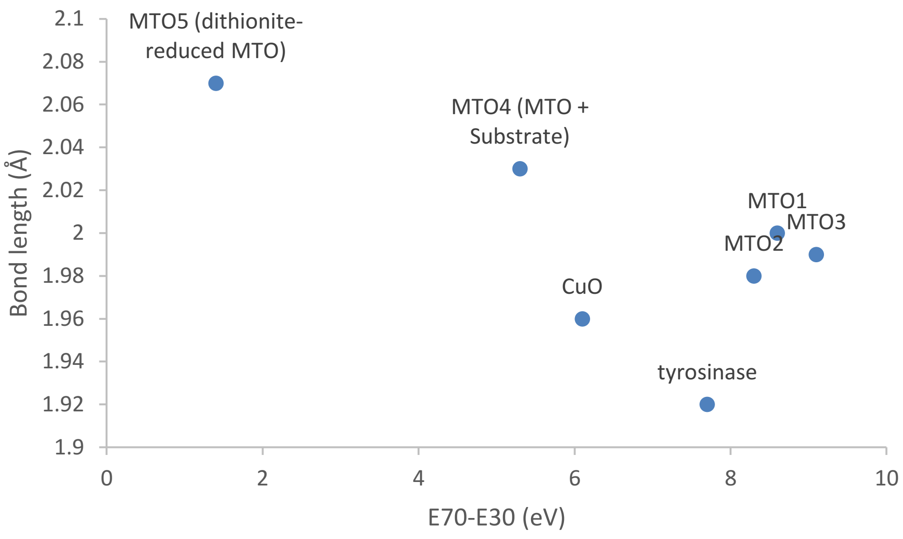

Supplementary Figure S7. Correlation between the copper-light atom bond length and E_{70}-E_{30} (which is a proxy for the ionisation energy of the copper, as detailed in the text).

>MT-oxidase Hyphomicrobium VS

MKKHLLAGACALAMGFAVIPGTFADETCNSPFTTALITGQEQYLHVWTLGMPGVGDESDK
LVTISVDPKSDKYGKVINTLSVGGRGEA**HH**TGFTDDRRYLWAGRLDDNKIFIFDLIDPAN
PKLIKTITDFADRTGYVGP**H**TFYALPGRMLIQALSNTKTHDGQTGLAVYSNAGELVSLHP
MPVTDGGDGYGYDIGINPAKNVLLTSSFTGWNNYMMDLGKMVKDPEAMKRFGNTMAIWDL
KSMKAEKILNVPGAPLEIRWSLKPEHNWAYTATALTSKLWLIKQDDKGEWIAKETGTIGD
PSKIPLPVDISITADAKGLWVNTFLDGTTRFYDISEPEHPKEVFSKKMGNQVNMVSQSYD
GKRVYFTTSLIANWDKKGAENDQWLKAYDWDGKELVEKFTVDFNELKLGRA**H**HMKFSSKT
NAAELGTNQSFPTRQ

Theoretical pI/Mw: 6.28 / 48306.93 Da
Theoretical pI/Mw: 5.97 / 45905.94 Da **+ 2 Cu + 4 Ca ➔ 46,193 Da**
(after removing the **Signal peptide**)

N-terminus
Peptides identified
Histidine residues potentially coordinating Cu

Purified MTO
➔ 46,186 Da

Supplementary Figure S8. Predicted amino acid sequence of the *Hyphomicrobium* sp. VS methanethiol oxidase with annotation of sequence features.

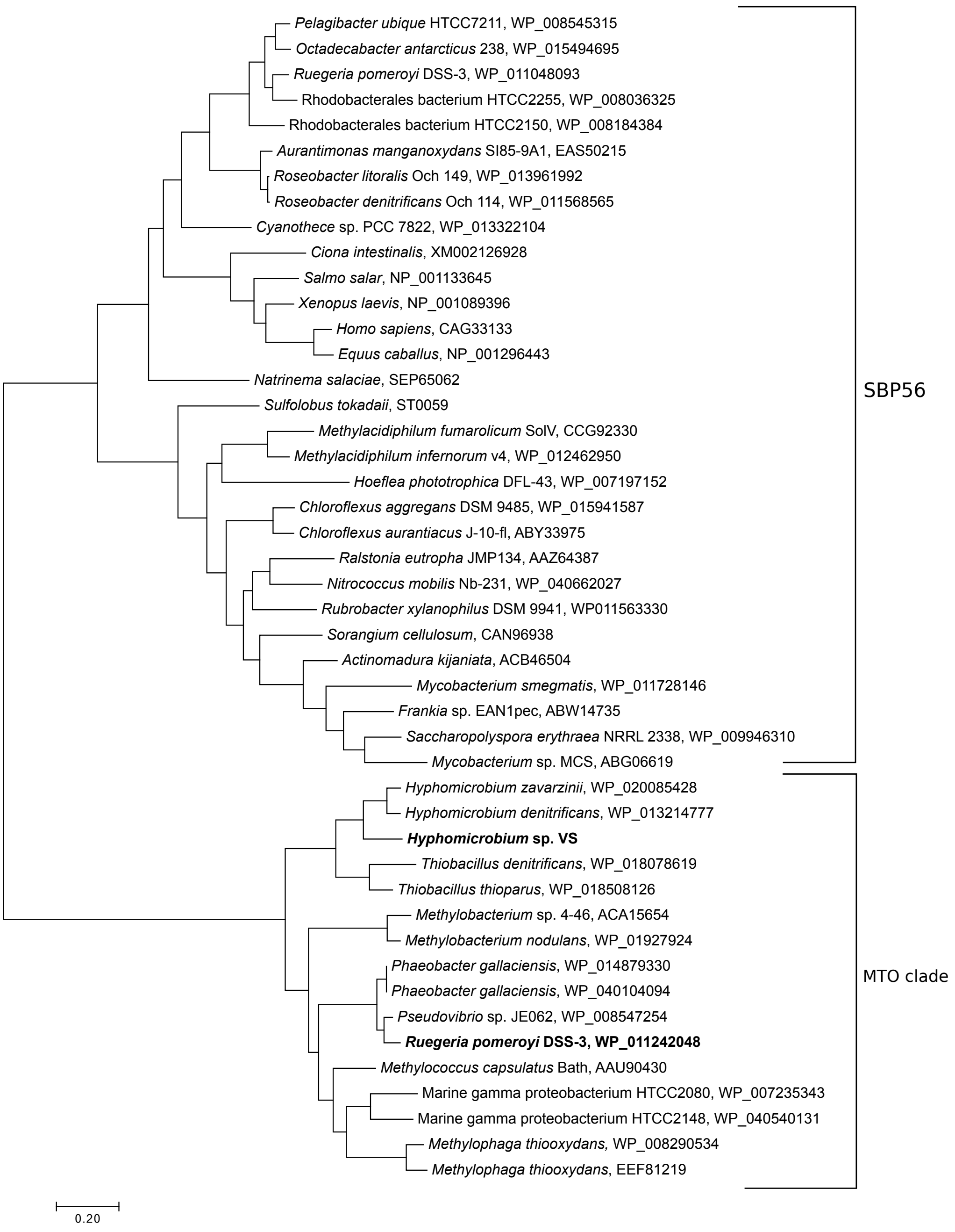

Supplementary Figure S9. Relationship of MtoX of *Hyphomicrobium* sp. VS, *Ruegeria pomeroyi* DSS-3 and other bacteria to those of selected representatives of the SBP56 protein family. The tree was inferred by using the Maximum Likelihood method based on the JTT matrix-based model (Jones et al 1992). The tree with the highest log likelihood (-16193.5993) is shown. Initial tree(s) for the heuristic search were obtained automatically by applying Neighbor-Join and BioNJ algorithms to a matrix of pairwise distances estimated using a JTT model, and then selecting the topology with superior log likelihood value. The tree is drawn to scale, with branch lengths measured in the number of substitutions per site. The analysis involved 47 amino acid sequences. All positions containing gaps and missing data were eliminated. There were a total of 356 positions in the final dataset. Evolutionary analyses were conducted in MEGA7 (Kumar et al 2016).

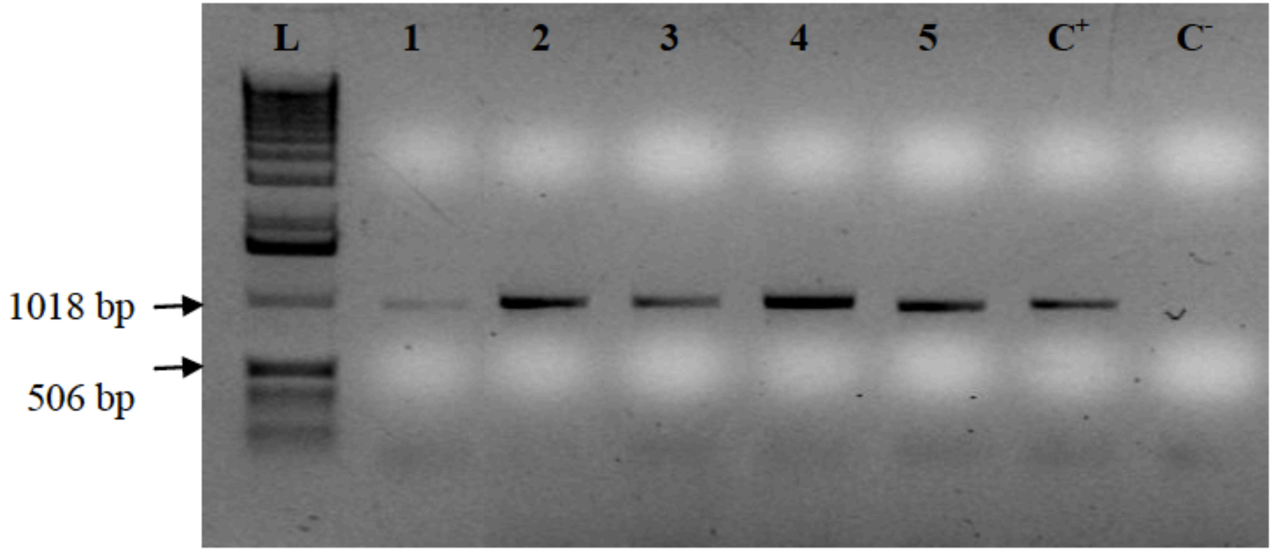

Supplementary Figure S10. Negative image of agarose gel electrophoresis analysis of PCR products obtained using *mtoX* specific primers 44F1+2 and 370R1+2+3. L: 1 kb ladder; 1 *Hyphomicrobium denitrificans*; 2 *Phaeobacter gallaeciensis*; 3 *Pseudovibrio ascidiaceicola*; 4 *Methylococcus capsulatus* (Bath); 5 *Methylocystis* sp. ATCC49242; C^+ positive control with *Methylophaga thiooxydans* DNA; C^-, no template control.

Supplementary Table S3. *Hyphomicrobium* strain VS, draft genome information

Sequence length	3,722,323 bases
GC %	59.2 %
Number of Contigs	347
Average contig length	9125 bp
Average coverage	480 x
Average CDS length	853 bp
Average intergenic length	241 bp
Protein coding density	88.86 %
Number of CDS	3598
misc_RNA	9
tRNA	46
ribosomal RNA 16s_rRNA	1
ribosomal RNA 23s_rRNA	1
ribosomal RNA 5s_rRNA	1

Supplementary Table S4. Results of testing of individual bacterial strains for MT degradation and amplification of *mtoX* genes with PCR primers 44F1/2 and 370R1/2/3

Strain name	MT degradation	*mto* amplification
Methylococcus capsulatus Bath	+	+
Hyphomicrobium denitrificans DSM1869	+	+
Hyphomicrobium sp. strain Bras 1	+	+
Hyphomicrobium sp. strain Bras 3	+	+
Hyphomicrobium sp. strain Moss 2	+	+
Methylobacterium chloromethanicum CM4	-	-
Methylobacterium dichloromethanicum DM4	-	-
Methylobacterium extorquens PA1	-	-
Methylobacterium extorquens AM1	-	-
Methylobacterium radiotolerans JCM2831	-	-
Methylobacterium populi BJ001	-	-
Methylophaga thiooxydans DMS010	+	+
Methylophaga marina DSM 5689	-	-
Methylocystis sp. ATCC49242	+	+
Thiobacillus thioparus TK-m	+	+
Thiobacillus thioparus E6	+	+
Phaeobacter gallaeciensis DSM 17395	+	+
Pseudovibrio ascidiaceicola DSM 16392	+	+

Supplementary Table S5. Primers for amplification of *mtoX*

Primer name	Conserved amino acid sequence motif	Primer sequence 5'-3'
44F1	VYVWTLG	GTY TAY GTY TGG ACN CTN GG
44F2	VYVWTLG	GTY TAY GTY TGG ACN TTA GG
44F3	LHVWTLG	CTY CAY GTG TGG ACR CTY GG
44F4	VYIWTLG	GTT TAT ATC TGG ACC TTG GG
352R1	NMVSSSW	TCC CAR CTY GAR CTS ACC AT
352R2	NMVSSSY	TCA TAG GAY GAN GAS ACC AT
352R3	NMVSSQSF	TCG AAG GAY GAN GAS ACC AT
352R4	NMVSSQSW	TCC CAR CTY TGR CTS ACC AT
370R1	LANWDKK	CYT TYT TRT CCC ART TNG CNA
370R2	LANWDHK	CYT TRT GRT CCC ART TNG CNA
370R3	LANWDKT	CSG TYT TRT CCC ART TNG CNA
MtoX41Fmodv2inos	(V/L)(Y/H)VWTLG	STY YAY GTY TGG ACI YTI GG
MTOX352Rmod	LANWD(H/K)(K/T)	CNK TNT KRT CCC ART TNG CNA

Supplementary Table S6. Metal content of MTO protein fractions with different subunit MW

	Metal content as atoms per MTO tetramer		
Element	Fraction 18 (lower band)	Fraction 19 (upper and lower band)	Fraction 20 (upper band)
Ca	3.47	n.d.	n.d.
Cu	1.44	1.44	n.d.
Fe	n.d.	n.d.	n.d.
Se	n.d.	n.d.	n.d.

Footnotes:
Compare fraction numbers with SDS-PAGE image in Supplementary Fig. S2.
n.d. not detected or trace levels only

Table S7. Genomes in IMG (img.jgi.doe.gov) with *mtoX* homologs and its genomic context including presence of genes encoding SCO1/SenC, MauG domains

Locus Tag of *mtoX*	Genome	SCO1/SenC domain (COG1999)	MauG domain (COG1858)	SCO1/MauG fusion?	other genes potentially in operon (or further up- or downstream)
K257DRAFT_3283	Alteromonadaceae bacterium 2141T.STBD.0c.01a	K257DRAFT_3284	K257DRAFT_3284	yes	Peroxiredoxin
K257DRAFT_3389	Alteromonadaceae bacterium 2141T.STBD.0c.01a	K257DRAFT_3390			-
P897DRAFT_3411	Alteromonadaceae bacterium 2719K.STB50.0a.01	P897DRAFT_3410	P897DRAFT_3410	yes	Peroxiredoxin
BegalDRAFT_0593	Beggiatoa alba B18LD	-	-		hypothetical, signal transduction histidine kinase
Ga0077526_11461	Betaproteobacteria sp. genome_bin_9 Ga0077526	Ga0077526_11462	Ga0077526_11463		-
A3AQDRAFT_04097	Bradyrhizobium japonicum USDA 4	A3AQDRAFT_4096	A3AQDRAFT_4112		S-(hydroxymethyl)glutathione DH and S-(hydroxymethyl)glutathione synthase in upstream region
BurJ1DRAFT_0065	Burkholderiales sp. JOSHI_001	BurJ1DRAFT_0066	BurJ1DRAFT_0067		-
DAMO_0405	Candidatus Methylomirabilis oxyfera sp. Australia	DAMO_0403	-		-
K320DRAFT_01023	Comamonas badia DSM 17552	K320DRAFT_01024	K320DRAFT_01025		anti ECF sigma factor, S-(hydroxymethyl)glutathione dehydrogenase / alcohol dehydrogenase
TE1149DRAFT_02382	Composite genome from Trout Bog Epilimnion pan-assembly TBepi.metabat.1149	TE1149DRAFT_02383	TE1149DRAFT_02383	yes	-
TE5136DRAFT_00864	Composite genome from Trout Bog Epilimnion pan-assembly TBepi.metabat.5136	TE5136DRAFT_00865	TE5136DRAFT_00865	yes	-
TH02062DRAFT_01233	Composite genome from Trout Bog Hypolimnion pan-assembly TBhypo.metabat.2062.v2	TH02062DRAFT_01234	TH02062DRAFT_01234	yes	type I restriction enzyme system, TonB receptor, ABC transporter, HlyD transporter protein
NSS_00025510	Dechloromarinus chlorophilus NSS	NSS_00025500 and NSS_00025490	NSS_00025500	yes	second Sco1 (Sco1/2), TetR (divergent upstream), downstream: S-(hydroxymethyl)glutathione dehydrogenase / alcohol dehydrogenase, S-formylglutathione hydrolase
G505DRAFT_01877	Ferrimonas futtsuensis DSM 18154	G505DRAFT_01878	-		hypothetical, enonuclease, LysR (could be separate from operon)
H598DRAFT_00632	Ferrimonas kyonanensis DSM 18153	H598DRAFT_00631	-		hypothetical, enonuclease, hypothetical, endoculcease, hypotehtical, alpha/beta hydrolase, LysR (could be separate from operon)
Ga0074792_114101	Ferrimonas sediminum DSM 23317	Ga0074792_114100	-		Alpha/beta hydrolase family protein; 2 hypotheticals; DNA-binding transcriptional regulator, LysR family
Ga0077536_1232	Gammaproteobacteria sp. genome_bin_26 Ga0077536	Ga0077536_1233	Ga0077536_1233	yes	three hypotheticals
Ga0056856_14213	Haloferula sp. BvORR071	Ga0056856_14212	-		Organic hydroperoxide reductase OsmC/OhrA, Acetyltransferase (GNAT) domain-containing protein, PAS domain S-box-containing protein (upstream,likely not same operon)
Hden_0743	Hyphomicrobium denitrificans ATCC 51888	Hden_0742	Hden_0741		LysR type regulator (divergently transcribed upstream)
HypVSv1_1800007	Hyphomicrobium sp. VS (mto cluster NCBI accession: KY242492)	downstream of *mtoX*	downstream of SCO1/SenC		-
F812DRAFT_1881	Hyphomicrobium zavarzinii ATCC 27496	F812DRAFT_1882	F812DRAFT_1883		sox operon downstream with soxYZBC, cyt553, cytCbb3, fccB, sulfide:quinone oxidoreductase
JANGC3DRAFT_1114	Janthinobacterium sp. CG3	JANGC3DRAFT_1115	-		-
Jab_2c22450	Janthinobacterium sp. HH01	Jab_2c22440			hemoglobin/transferrin/lactoferrin receptor protein, putative hemin transport protein, polyketide cylcase dehydrase
Leina_04065	Leisingera nanhaiensis NH52F, DSM 24252 (scaffold version)	Leina_04066	Leina_04067		IclR, hypothetical
LeumuDRAFT_1776	Leucothrix mucor DSM 2157	LeumuDRAFT_1777	LeumuDRAFT_1777	yes	S-(hydroxymethyl)glutathione DH and S-formylglutathione hydrolase, hypothetical, ATPase component of ATP transporter, hypothetical
Ga0072465_11476	Magnetospira sp. QH-2	Ga0072465_11477	Ga0072465_11478		S-(hydroxymethyl)glutathione synthase
MGP2080_08771	Marine gamma proteobacterium sp. HTCC2080	MGP2080_08776	MGP2080_08766		succinate semialdehyde DH
GPB2148_3671	Marine gamma proteobacterium sp. HTCC2148	GPB2148_3716	GPB2148_3751		sulfide quinone oxidoreductase
MetluDRAFT_3319	Methylobacter luteus IMV-B-3098	MetluDRAFT_3320	MetluDRAFT_3320	yes	TetR, DUF4242
EK22DRAFT_03406	Methylobacter sp. BBA5.1	EK22DRAFT_03407	EK22DRAFT_03407	yes	TetR, DUF4242

Supplementary Table S7 (continued)

Locus Tag of *mtoX*	Genome	SCO1/SenC domain (COG1999)	MauG domain (COG1858)	SCO1/MauG fusion?	other genes potentially in operon (or further up- or downstream)
GY38DRAFT_0234	Methylobacter whittenburyi ACM 3310	GY38DRAFT_0233	GY38DRAFT_0233	yes	TetR, DUF4242
MetmaDRAFT_2041	Methylobacterium marinus A45	MetmaDRAFT_2042	MetmaDRAFT_2042	yes	TetR, DUF4242
Mnod_1226	Methylobacterium nodulans ORS 2060	Mnod_1225	-		dimethylmenaquinone transferase,
FF93DRAFT_05962	Methylobacterium sp. 174MFSha1.1	FF93DRAFT_05961	-		adenylate cyclase
M446_1126	Methylobacterium sp. 4-46	M446_1125	-		OMP barrel transporter, peptidoglycan binding, glycoside hydrolase
MET2598DRAFT_04480	Methylobacterium sp. WSM2598	MET2598DRAFT_04481	-		ion channel, OMP barrel, 1,4 alpha glucan branching eenzyme
Ga0080943_107828	Methylobacterium tarhaniae DSM 25844	Ga0080943_107829			Adenylate cyclase, class 3
Ga0080942_115226	Methylobacterium variabile DSM 16961	Ga0080942_115225	-		Adenylate cyclase, class 3
JC06DRAFT_3718	Methylocaldum sp. 175	-	-		FeS cluster protein YjdI, DUF4242
MetszDRAFT_2121	Methylocaldum szegediense O-12	-	-		TetR, DUF4242, methionyl-tRNAsynthetase
MetacDRAFT_2724	Methylocapsa acidiphila B2	MetacDRAFT_2723 (SCO1/2)	-		formate DH subunit upstream
EK23DRAFT_02364	Methylococaceae sp. 73a	EK23DRAFT_02365	EK23DRAFT_02365	yes	TetR, DUF4242
Ga0078419_138204	Methylococcaceae sp. B42 Ga0078419	-	-		TetR, DUF4242, PAS domain S-box-containing protein/diguanylate cyclase (GGDEF) domain-containing protein;
Ga0081636_13963	Methylococcaceae-55 (UID203)	Ga0081636_13962	Ga0081636_13962	yes	TetR, DUF4242
MCA0317	Methylococcus capsulatus Bath	MCA0318	MCA0318	Yes	TetR, hypothetical
O5ODRAFT_03774	Methylocystis parvus OBBP	O5ODRAFT_03775	O5ODRAFT_03776		TetR, DUF4242, peptidyl-dipeptidase Dcp, Cytochrome c553
A3OODRAFT_1465	Methylocystis rosea SV97T	A3OODRAFT_1466	A3OODRAFT_1467		TetR, DUF4242
Met49242_2344	Methylocystis sp. Rockwell, ATCC 49242	Met49242_2345	Met49242_2346		TetR, tRNA MetCAT
SB2_02768	Methylocystis sp. SB2	SB2_02767	SB2_02766		TetR, DUF4242; downstream, likely other operon: ribonuclease P protein component, protein translocase subunit, GTP binding prot., N-acetylglutamate kinase, NAD-dependent deacetylase, putative hydrolase of HAD family
BN69_2794	Methylocystis sp. SC2	BN69_2795	BN69_2796		TetR, DUF4242, rpmH, rnpA, oxaA, engB GTP binding protein, N-acetylglutamate kinase, NAD-dependent deacetylase, putative hydrolase of the HAD superfamily, DUF4238
MKO_00251	Methyloglobulus morosus KoM1	-	-		tetR, DUF4242, uncharacterised FeS cluster protein YjdI, methylene-tetrahydromethanopterin dehydrogenase
H035DRAFT_1461	Methylohalobius crimeensis 10Ki	-	-		multicopper oxidase, GTP cyclohydrolase II, epoxyquoesine reductase, PAS domain S-box-cont. prot./diguanylate cyclase (GGDEF) domain-cont. prot., methyltransferase domain cont. prot., sulfotransferase domain cont. prot.
EP25DRAFT_2710	Methylomarinum vadi IT-4	EP25DRAFT_2709	EP25DRAFT_2709	yes	TetR, DUF4242, hypothetical, OmpA
CC94DRAFT_2098	Methylomicrobium agile ATCC 35068	-	-		TetR, DUF4242, 4Fe-4S mono cluster
MetalDRAFT_0731	Methylomicrobium album BG8	-	-		TetR, DUF4242, hypothetical
MEALZ_0120	Methylomicrobium alcaliphilum 20Z	MEALZ_0119	MEALZ_0119		TetR, DUF4242
METBUDRAFT_2991	Methylomicrobium buryatense 5G	METBUDRAFT_2992	METBUDRAFT_2992	yes	TetR, DUF4242
IQ34DRAFT_2204	Methylomicrobium kenyense AMO1	IQ34DRAFT_2205	IQ34DRAFT_2205	yes	TetR, DUF4242
Metme_00042630	Methylomonas methanica MC09	-	-		HlyD family secretion protein, ABC transporter ATP binding component, TetR, DUF4242
Meth11bDRAFT_2463	Methylomonas sp. 11b	Meth11bDRAFT_2459	Meth11bDRAFT_2459		TetR, DUF4242, hypothetical genes

Supplementary Table S7 (continued)

Locus Tag of *mtoX*	Genome	SCO1/SenC domain (COG1999)	MauG domain (COG1858)	SCO1/MauG fusion?	other genes potentially in operon (or further up- or downstream)
U737DRAFT_00751	Methylomonas sp. LW13	U737DRAFT_00753	U737DRAFT_00753	yes	DUF4242, DUF4437, phoB, phoR
G006DRAFT_3546	Methylomonas sp. MK1	G006DRAFT_3551	G006DRAFT_3551	yes	TetR, addiction module, DUF4242, plasmid stabilisation ParE, antitoxin type II, hypotheticals
Ga0070578_101243	Methylophaga sulfidovorans DSM 11578	Ga0070578_101244	Ga0070578_101244	yes	Peroxiredoxin, Glyoxylase, beta-lactamase superfamily II (Rhodanese domain), sulfide:quinone oxidoreductase, sulfate permease, SulP family
MDMS009_211	Methylophaga thiooxydans DMS010	MDMS009_51	MDMS009_51	Yes	sulfide quinone oxidoreductase, rhodanese-like domain S transferase, ion trabnsporter SulP family, adenylylsulfate kinase
MDMS009_768	Methylophaga thiooxydans DMS010	MDMS009_908	MDMS009_908	Yes	sulfide quinone oxidoreductase, rhodanese-like domain S transferase, ion trabnsporter SulP family
A3OWDRAFT_1480	Methylosarcina fibrata AML-C10	-	-		TetR, DUF4242, hypothetical
MetlaDRAFT_3465	Methylosarcina lacus LW14	-	-		TetR, hypothetical, subtilase family protein, sulfoxide reducatse catalytic subunit YedY, sulfite DH cytochrome subunit, hypothetical
Ga0081643_121524	Methylosarcina lacus-69 (UID4274)	-	-	-	TetR, DUF4242, hypothetical
Ga0081622_12747	Methylosarcina-21 (UID203)	-	-	-	TetR, DUF4242, hypothetical
MetFAM1DRAFT_2737	Methyloversatilis sp. FAM1	MetFAM1DRAFT_2738	MetFAM1DRAFT_2739		dCTP deaminase, PEP-CTERM protein-sorting domain-containing protein, arginine decarboxylase
MetRZ18153DRAFT_0157	Methyloversatilis sp. RZ18-153	MetRZ18153DRAFT_0156	MetRZ18153DRAFT_0155		dCTP deaminase, PEP-CTERM protein-sorting domain-containing protein, arginine decarboxylase
MicloDRAFT_00013950	Microvirga lotononidis WSM3557	MicloDRAFT_00013940			FAD/FMN containing DH, hypothetical, SAM (sterial alpha motif) domain
U745DRAFT_0655	Oceanicola sp. MCTG156(1a)	U745DRAFT_0656	U745DRAFT_0657		IclR, methyltransferase domain containing protein, Predicted flavoprotein CzcO associated with the cation diffusion facilitator CzcD
PGA2_c06660	Phaeobacter gallaeciensis 2.10	PGA2_c06650	PGA2_c06640		IclR
RGBS107_10636	Phaeobacter gallaeciensis BS107	RGBS107_10631	RGBS107_10626		IclR
Gal_02776	Phaeobacter gallaeciensis DSM 26640	Gal_02777	Gal_02778		IclR
Phain_00699	Phaeobacter inhibens T5, DSM 16374	Phain_00698	Phain_00697		IclR
Ga0056025_01449	Pseudoalteromonas denitrificans DSM 6059	Ga0056025_01450	Ga0056025_01450		peroxiredoxin
Ga0070514_11458	Pseudovibrio ascidiaceicola DSM 16392	Ga0070514_11457	Ga0070514_11456	-	IclR
PSE_2928	Pseudovibrio sp. FO-BEG1	PSE_2927	PSE_2926		IclR
PJE062_1794	Pseudovibrio sp. JE062	PJE062_1508	PJE062_1164		IclR
Ga0079812_0793	Pseudovibrio sp. Tun.PHSC04-5.I4	Ga0079812_0792	Ga0079812_0791		IclR
B594DRAFT_0559	Rhodocyclaceae bacterium RZ94	B594DRAFT_0560	B594DRAFT_0561		dCTP deaminase, PEP-CTERM protein-sorting domain-containing protein, arginine decarboxylase
TRICHSKD4_5563	Roseibium sp. TrichSKD4	TRICHSKD4_5564	TRICHSKD4_5565		hypothetical
SPOA0269	Ruegeria pomeroyi DSS-3	SPOA0270	SPOA0271		IclR, glutathione dependent formaldehyde dehydrogenase
Ga0052890_03493	Rugamonas rubra ATCC 43154	Ga0052890_03492			DUF2892, alkylhydroperoxidase AhpD, Ca2+-transporting ATPase, cation:H+ antiporter
A3GODRAFT_01888	Sedimenticola selenatireducens DSM 17993	A3GODRAFT_01889	A3GODRAFT_01889	yes	peroxiredoxin
A3GODRAFT_00092	Sedimenticola selenatireducens DSM 17993	A3GODRAFT_00091	A3GODRAFT_00091	yes	Sco1, S-(hydroxymethyl)glutathione DH, S-(hydroxymethylglutathione hydrolase, hypothetical, TetR upstream divergent
CUZ_03126	Sedimenticola sp. CUZ	CUZ_03125	CUZ_03125	yes	Sco1, S-(hydroxymethyl)glutathione DH, S-(hydroxymethylglutathione hydrolase, hypothetical, TetR upstream divergent
CUZ_01043	Sedimenticola sp. CUZ	CUZ_01042	CUZ_01042	yes	peroxiredoxin

Supplementary Table S7 (continued)

Locus Tag of *mtoX*	Genome	SCO1/SenC domain (COG1999)	MauG domain (COG1858)	SCO1/MauG fusion?	other genes potentially in operon (or further up- or downstream)
Ga0081741_112291	Sedimenticola sp. SIP-G1	Ga0081741_112290	Ga0081741_112290		peroxiredoxin
Ga0069263_11326	Sedimenticola thiotaurini SIP-G1	Ga0069263_11327	Ga0069263_11327	yes	peroxiredoxin
Ga0069205_1003242	Skermanella aerolata KACC 11604	Ga0069205_1003243	-	-	TetR, 2-polyprenyl-6-methoxyphenol hydroxylase, cation/acetate symporter, acetolactate synthase-1/2/3, Cytochrome c, mono- & diheme variants, Aerobic-type CO dehydrogenase (DH, small subunit, CoxS/CutS family, CO or xanthine DH Mo-binding subunit, PAS domain S-box-cont. prot., nickel transport system
N825_12395	Skermanella stibiiresistens SB22	N825_12400	-	-	Ubiquinone/menaquinone biosynthesis C-methylase UbiE (methyltransferase type 11)
F574DRAFT2856	Thioalkalivibrio sp. AL5	F574DRAFT2857	F574DRAFT2858	yes, 2857	peroxiredoxin; further downstream, probably not part of operon: cytochrome subunit of sulfide dehydrogenase, FAD(NAD) dependent DH (probably partial fccB)
F468DRAFT_02573	Thioalkalivibrio sp. ALJ2	F468DRAFT_02574	F468DRAFT_02575	yes, 2574	peroxiredoxin; further downstream, probably not part of operon: cytochrome subunit of sulfide dehydrogenase, full fccB
C936DRAFT_2731	Thioalkalivibrio sp. ALJ4	C936DRAFT_2730	C936DRAFT_2729	yes, 2730	peroxiredoxin; further downstream, probably not part of operon: cytochrome subunit of sulfide dehydrogenase, FAD(NAD) dependent DH (probably partial fccB)
C937DRAFT_2571	Thioalkalivibrio sp. ALJ5	C937DRAFT_2572	C937DRAFT_2573	yes, 2572	peroxiredoxin; further downstream, probably not part of operon: cytochrome subunit of sulfide dehydrogenase, FAD(NAD) dependent DH (probably partial fccB)
G317DRAFT_2745	Thioalkalivibrio sp. ALJ9	G317DRAFT_2744	G317DRAFT_2743	yes, 2744	peroxiredoxin; further downstream, probably not part of operon: cytochrome subunit of sulfide dehydrogenase, FAD(NAD) dependent DH (probably partial fccB)
F465DRAFT_2840	Thioalkalivibrio sp. ARh4	F465DRAFT_2841	F465DRAFT_2842	yes, 2841	peroxiredoxin; further downstream, probably not part of operon: cytochrome subunit of sulfide dehydrogenase, FAD(NAD) dependent DH (probably partial fccB)
C164DRAFT_2790	Thioalkalivibrio versutus AL2	C164DRAFT_2789	C164DRAFT_2788	yes, 2789	2nd MauG, peroxireductase, sulfide DH fccAB
B059DRAFT_02518	Thiobacillus denitrificans DSM 12475	B059DRAFT_02517	B059DRAFT_02516		LysR cys regulon activator (upstream), formaldehyde activating enzyme, alkane monooxygenase, hemoglobin
B058DRAFT_01665	Thiobacillus thioparus DSM 505	B058DRAFT_01664	B058DRAFT_01663		LysR cys regulon activator (upstream), formaldehyde activating enzyme, FeS cluster protein YjdI, NADH DH (FAD subunit), Rieske FeS protein,
ThidrDRAFT_3297	Thiorhodococcus drewsii AZ1	ThidrDRAFT_3298	ThidrDRAFT_3298, ThidrDRAFT_3299	yes	2nd copy of MauG, Threonine/homoserine efflux transporter RhtA downstream
A3IEDRAFT_00868	Thiothrix disciformis DSM 14473	A3IEDRAFT_00869	A3IEDRAFT_00869	yes	A3IEDRAFT_00868 is a Sco1/SenC/PrrC, tyrosine phosphatase
Ga0097622_102911	unclassified betaproteobacterium ENR4	Ga0097622_102910	Ga0097622_10299		-
G346DRAFT_1491	Verrucomicrobium sp. LP2A	G346DRAFT_1490	-		phosphoadenylylsulfate reductase (thioredoxin), 3-octaprenyl-4hydroxybenzoate decarboxylase, UDP-glucuronate 4-epimerase, Uncharacterized conserved protein YecE, DUF72 family, 3 hypotheticals

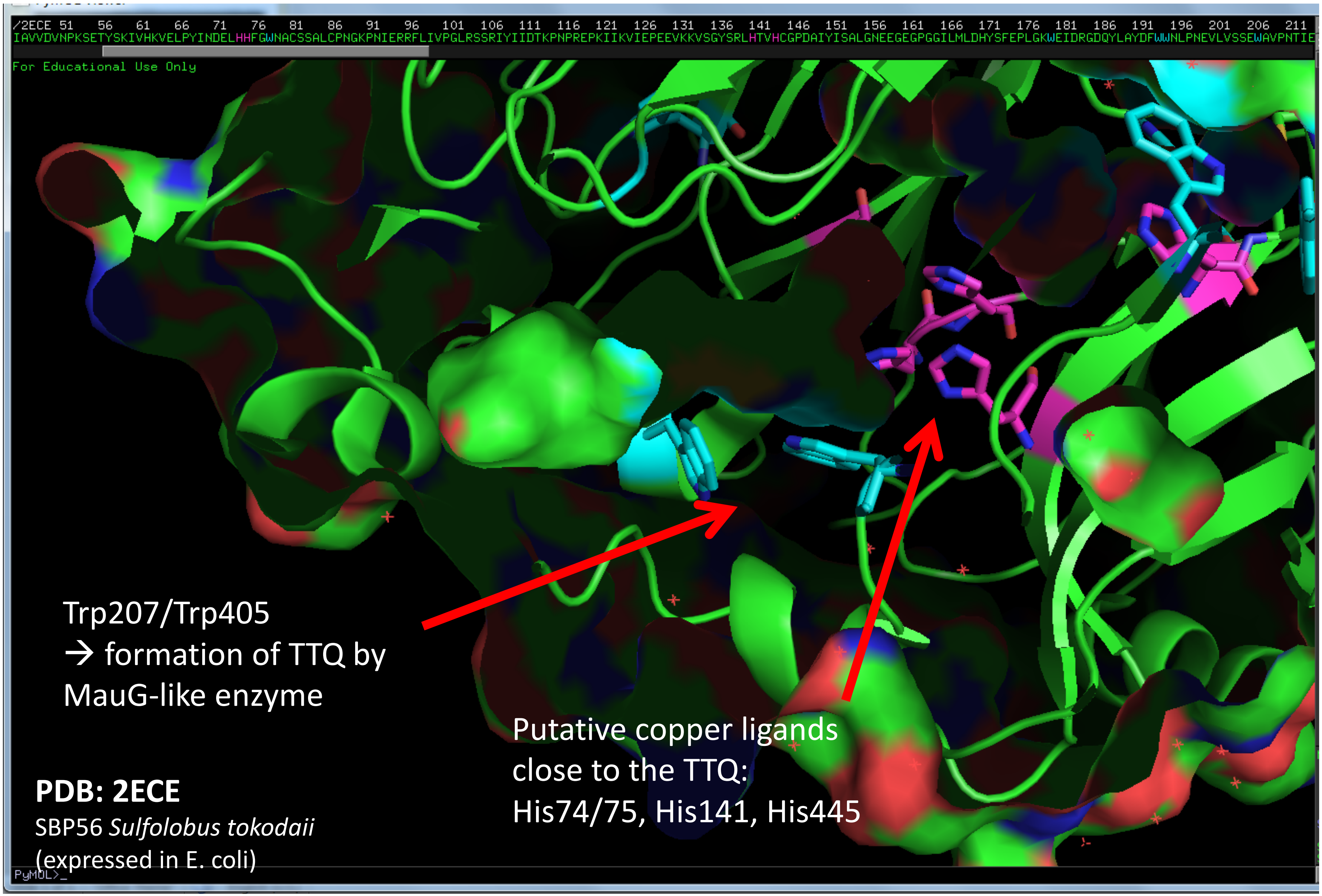

Supplementary Figure S11. Structural detail of SPB56 from *Sulfolobus tokodaii* (PDB accession: 2ECE), showing tryptophan residues that would form putative TTQ cofactor upon maturation by MauG-like enzyme and putative copper ligands. See main text and EXAFS supplementary information for more detail.

	W211 (top) / W207 (bottom)	W374 (top) / W405 (bottom)
Hyphomicrobium VS	TG**W**NNY	YFTTSLIAN**W**D
Homo sapiens SBP56	TE**W**AAP	YITTSLYSA**W**D
Methylacidiphilum fumariolicum SolV	SE**W**GTP	YLTNSLYWA**W**D
Sulfolobus tokodaii st0059	SE**W**AVP	YVTNSLYST**W**D

	H89/90 (top) / H74/75 (bottom)	H140 (top) / H141 (bottom)	H412 (top) / H449 (bottom)
Hyphomicrobium VS	GEA**HH**TGFTD	GYVGP**H**TFYA	RA**H**HMKFS
Homo sapiens SBP56	DEL**HH**SGWNT	ELAFL**H**TSHC	LA**H**ELRYP
Methylacidiphilum fumariolicum SolV	DEL**HH**YGWNA	GYSRP**H**TVHC	RP**H**QVRLQ
Sulfolobus tokodaii st0059	DEL**HH**FGWNA	GYSRL**H**TVHC	RS**H**QVRLS

Supplementary Figure S12. Conservation of the W residues potentially involved in formation of a TTQ cofactor by MauG and putative copper ligands in *mtoX* of *Hyphomicrobium* sp. VS and homologues in *Methylacidiphilum fumarolicum*, *Sulfolobus tokodaii* and the human SBP56 homologue *SELENBP1.*

References

Andersson KK, Schmidt PP, Katterle B, Strand KR, Palmer AE, Lee S-K *et al* (2003). Examples of high-frequency EPR studies in bioinorganic chemistry. *J Biol Inorg Chem* **8:** 235-247.

Antholine WE, Kastrau DHW, Steffens GCM, Buse G, Zumft WG, Kroneck PMH (1992). A comparative EPR investigation of the multicopper proteins nitrous-oxide reductase and cytochrome-*c*-oxidase. *Eur J Biochem* **209:** 875-881.

Chauhan S, Kline CD, Mayfield M, Blackburn NJ (2014). Binding of copper and silver to single-site variants of peptidylglycine monooxygenase reveals the structure and chemistry of the individual metal centers. *Biochemistry* **53**: 1069-1080.

Gurman SJ, Binsted N, Ross I (1986). A rapid, exact, curved-wave theory for EXAFS calculations. ii. the multiple-scattering contributions. *J Phys C: Solid State* **19**: 1845-1861.

Jones DT, Taylor WR, Thornton JM (1992). The rapid generation of mutation data matrices from protein sequences. *Comp Appl Biosci* **8**: 275-282.

Kaim W, Schwederski B, Klein A (2013). *Bioinorganic Chemistry - Inorganic Elements in the Chemistry of Life: An Introduction and Guide*, 2nd Edition edn, vol. 2nd Edition.

Kumar S, Stecher G, Tamura K (2016). MEGA7: Molecular Evolutionary Genetics Analysis version 7.0 for bigger datasets. *Mol Biol Evol* **33:** 1870-1874.

Monzani E, Quinti L, Perotti A, Casella L, Gullotti M, Randaccio L *et al* (1998). Tyrosinase models. Synthesis, structure, catechol oxidase activity, and phenol monooxygenase activity of a dinuclear copper complex derived from a triamino pentabenzimidazole ligand. *Inorg Chem* **37:** 553-562.

Solomon EI, Sundaram UM, Machonkin TE (1996). Multicopper oxidases and oxygenases. *Chem Rev* **96:** 2563-2606.

van der Helm D, Franks WA (1968). A variation of the square-pyramidal copper(II) surrounding. A possible copper interaction with tyrosine. *J Am Chem Soc* **90**: 5627-5629.
